# Low‐Intensity Focused Ultrasound‐Responsive Ferrite‐Encapsulated Nanoparticles for Atherosclerotic Plaque Neovascularization Theranostics

**DOI:** 10.1002/advs.202100850

**Published:** 2021-08-11

**Authors:** Jianting Yao, Zhuowen Yang, Liandi Huang, Chao Yang, Jianxin Wang, Yang Cao, Lan Hao, Liang Zhang, Jingqi Zhang, Pan Li, Zhigang Wang, Yang Sun, Haitao Ran

**Affiliations:** ^1^ Department of Ultrasound Chongqing Key Laboratory of Ultrasound Molecular Imaging The Second Affiliated Hospital of Chongqing Medical University Chongqing 400010 P. R. China; ^2^ Department of Cardiology The First Affiliated Hospital Cardiovascular Institute Harbin Medical University Harbin 150001 P. R. China; ^3^ State Key Laboratory of Ultrasound in Medicine and Engineering The Second Affiliated Hospital of Chongqing Medical University Chongqing 400010 P. R. China; ^4^ Department of Radiology Chongqing General Hospital University of Chinese Academy of Sciences Chongqing 400014 P. R. China; ^5^ Department of Ultrasound The First Affiliated Hospital of Harbin Medical University Harbin 150001 P. R. China

**Keywords:** manganese ferrite, plaque neovascularization, sonodynamic therapy, theranostic probe

## Abstract

Pathological angiogenesis is a crucial factor that causes atherosclerotic plaque rupture. Sinoporphyrin sodium‐mediated sonodynamic therapy (DVDMS‐SDT) induces regression of plaque neovascularization in humans without causing obvious side effects. However, a clinical noninvasive theranostic strategy for atherosclerotic plaque neovascularization is urgently needed. A nanoplatform designed for multimodality imaging‐guided SDT in plaque angiogenesis theranostics, termed PFP–HMME@PLGA/MnFe_2_O_4_–ramucirumab nanoparticles (PHPMR NPs), is fabricated. It encapsulates manganese ferrite (MnFe_2_O_4_), hematoporphyrin monomethyl ether (HMME), and perfluoropentane (PFP) stabilized by polylactic acid‐glycolic acid (PLGA) shells and is conjugated to an anti‐VEGFR‐2 antibody. With excellent magnetic resonance imaging (MRI)/photoacoustic/ultrasound imaging ability, the distribution of PHPMR NPs in plaque can be observed in real time. Additionally, they actively accumulate in the mitochondria of rabbit aortic endothelial cells (RAECs), and the PHPMR NP‐mediated SDT promotes mitochondrial‐caspase apoptosis via the production of reactive oxygen species and inhibits the proliferation, migration, and tubulogenesis of RAECs. On day 3, PHPMR NP‐mediated SDT induces apoptosis in neovessel endothelial cells and improves hypoxia in the rabbit advanced plaque. On day 28, PHPMR NP‐mediated SDT reduces the density of neovessels, subsequently inhibiting intraplaque hemorrhage and inflammation and eventually stabilizing the plaque. Collectively, PHPMR NP‐mediated SDT presents a safe and effective theranostic strategy for inhibiting plaque angiogenesis.

## Graphical abstract

A clinical non‐invasive theranostic strategy for atherosclerotic plaque neovascularization is urgently needed. In this article, the strategy of “multi‐modality imaging‐guided SDT” to inhibit plaque neovascularization is proposed, which is more convenient for clinical use and makes SDT more accurate and efficient.

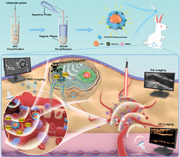

## 1 Introduction

Pathological angiogenesis is a crucial factor in promoting the rupture of vulnerable atherosclerotic plaques, leading to death and disability in humans.^[^ ^1^ ^]^ Hence, early detection and timely suppression of plaque angiogenesis presents a convincing strategy to prevent the occurrence of atherosclerosis‐associated adverse cardio‐cerebrovascular events.

Contrast‐enhanced ultrasound (US) imaging,^[^ ^2^ ^]^ dynamic contrast‐enhanced magnetic resonance imaging (MRI),^[^ ^3^ ^]^ and optical coherence tomography^[^ ^4^ ^]^ are the traditional imaging techniques used to detect plaque neovascularization. However, they are unable to assess plaque angiogenesis at the cellular and molecular levels. A previous study identified *α*_v_ *β*_3_‐targeted paramagnetic gadolinium nanoparticles (NPs) as potent molecular MRI T1 contrast agents; however, these agents pose an increased risk of renal systemic fibrosis in patients with renal insufficiency.^[^ ^5^ ^]^ Thus, biocompatible and low‐toxicity paramagnetic Fe_3_O_4_ nanoparticles were used for MRI/ positron emission tomography (PET) dual‐mode molecular imaging of plaque neovascularization in rabbits.^[^ ^6^ ^]^ Nevertheless, compared with the T1 enhancement contrast agent gadolinium, Fe_3_O_4_ crystals exhibited obviously insufficient spatial resolution and sensitivity in T2 negative imaging, and their contrast signal can be easily confused with bleeding, calcification, and artifacts.^[^ ^7^ ^]^ Therefore, a safe and sensitive T1 positive contrast agent for MRI molecular imaging is of great significance for the early detection of plaque neovascularization.

Metal spinel ferrite nanoparticles MFe_2_O_4_ (M = Mn^2+^, Fe^2+^, Co^2+^, Ni^2+^) represent an important class of magnetic ternary compound nanoparticles, which exhibit high chemical stability and controllable size and shape, primarily as highly sensitive MRI nanoprobes for in vivo and noninvasive detection of clinically important biological targets.^[^ ^8^ ^]^ Compared with FeFe_2_O_4_, CoFe_2_O_4_, and NiFe_2_O_4_, molecular probes based on MnFe_2_O_4_ nanoparticles, which have the strongest magnetic properties, exhibit considerably enhanced sensitivity for cancer detection without causing obvious toxicity.^[^ ^9^ ^]^ Moreover, MnFe_2_O_4_ can be safely and effectively used for MRI T1 positive imaging by reducing the size of the nanoprobe.^[^ ^10^ ^]^ Hence, we postulated that small‐scale MnFe_2_O_4_ nanoparticles could be an ideal contender for MRI detection of plaque neovessels.

Although MRI achieves acceptable soft tissue resolution and allows quantitative evaluation of atherosclerotic plaque components, its poor temporal resolution hampers its application in rapidly moving vessels.^[^ ^11^ ^]^ Photoacoustic imaging (PAI) is a hybrid imaging modality that merges optical illumination and ultrasound detection, and it is a highly sensitive technique that exhibits exceptional spatial resolution.^[^ ^12^ ^]^ US imaging is a noninvasive real‐time imaging technique that displays an acceptable penetration depth compared with PAI.^[^ ^13^ ^]^ Consequently, the use of multimodality imaging compensates for the disadvantages of MRI and synergizes its advantages, which is of great relevance for plaque neovascularization assessment.

Inorganic nanoparticles (such as MnO, CuS, etc.) have been widely used in the diagnosis and therapy of atherosclerosis.^[^ ^14^ ^]^ To date, antiangiogenic drugs and vascular endothelial growth factor‐A (VEGF‐A) blockers constitute the central therapeutic axis to suppress plaque neovascularization. However, antiangiogenic drugs display non‐negligible systemic toxicity, whereas systemic use of VEGF‐A inhibitors promotes significant hypertensive and thromboembolic events.^[^ ^15^ ^]^ Therefore, local therapeutic strategies may be a suitable alternative to safely prevent atherosclerotic plaque rupture. We previously demonstrated that sinoporphyrin sodium (DVDMS)‐mediated sonodynamic therapy (DVDMS‐SDT) inhibits plaque angiogenesis via macrophage apoptosis‐induced endothelial cell apoptosis, without causing obvious side effects.^[^ ^16^ ^]^ Nevertheless, its clinical application and therapeutic effect were limited by the lack of a technique for real‐time visualization of the distribution of sonosensitizers in vivo, and the inability of SDT to directly act on neovascular endothelial cells.

**Scheme 1:**
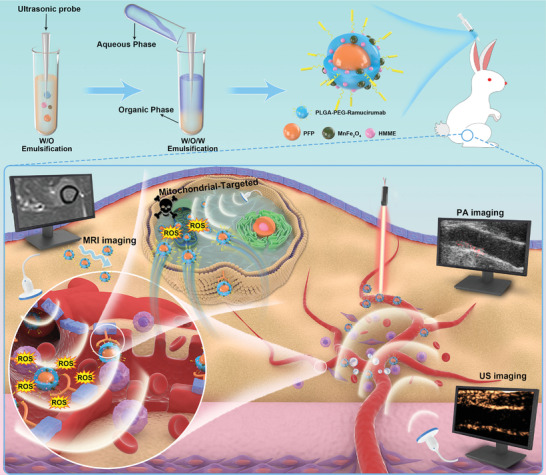
Schematic illustration of the synthetic process for PFP–HMME@PLGA/MnFe_2_O_4_–Ram nanoplatform and the corresponding theranostic functionality for targeted MRI/PA/US multimodal imaging‐guided sonodynamic plaque neovascularization therapy.

To optimize and monitor the efficacy of SDT in suppressing plaque neovascularization, we fabricated a nanoplatform that encapsulated 3 nm manganese ferrite (MnFe_2_O_4_), hematoporphyrin monomethyl ether (HMME), and perfluoropentane (PFP) stabilized by the nontoxic, degradable poly(lactic‐*co*‐glycolic acid) (PLGA) shells and conjugated at the surface to an anti‐VEGFR‐2 antibody to form ferrite‐loaded multifunctional nanoparticles (PFP–HMME@PLGA/MnFe_2_O_4_–ramucirumab). 3 nm MnFe_2_O_4_ was selected for MRI T1 and PA imaging. HMME, similar to DVDMS, is a porphyrin derivative and is a stable and safe sonosensitizer.^[^ ^17^ ^]^ PFP can be vaporized to gas microbubbles through the effect of acoustic droplet vaporization (ADV)^[^ ^18^ ^]^ and used for US molecular imaging. VEGF/VEGFR‐2 have unique roles in the mediation and promotion of intraplaque angiogenesis.^[^ ^19^ ^]^ Ramucirumab (Ram) is an FDA‐approved monoclonal antibody that binds to VEGFR‐2 to inactivate VEGF‐mediated downstream signaling pathways, leading to the inhibition of tumor neovascularization.^[^ ^20^ ^]^ The developed PFP–HMME@PLGA/MnFe_2_O_4_–ramucirumab nanoplatform could actively target the mitochondria of rabbit aortic endothelial cells (RAECs), increasing nanoparticle accumulation in plaque neovessels. Upon low‐intensity focused ultrasound (LIFU) irradiation, MRI/PA/US multimodal imaging in plaque‐bearing rabbits was conducted to provide real‐time observation of the biodistribution of the nanoplatform and provide guidance for plaque therapy. A multimodal imaging‐guided SDT was finally performed, which achieved effective and complete suppression of plaque neovascularization, resulting in the inhibition of intraplaque hemorrhage and inflammation, finally stabilizing the plaque (**Scheme** **1**). Therefore, this work offers an original strategy for early diagnosis, real‐time assessment, and effective treatment of atherosclerotic plaque neovascularization in the clinic.

## 2 Results and Discussion

### 2.1 Fabrication and Characterization of PFP–HMME@PLGA/MnFe_2_O_4_–Ram NPs

Paramagnetic oleic acid‐coated 3 nm MnFe_2_O_4_ exhibits wide absorption in the UV–vis range (Figures S1–S3, Supporting Information). As shown in Figure S4 in the Supporting Information, humanized ramucirumab effectively bound the RAECs. PFP, HMME, and MnFe_2_O_4_ were dissolved in an organic phase containing the polymer PLGA–PEG–ramucirumab (Figure S5, Supporting Information) to fabricate PFP–HMME@PLGA/MnFe_2_O_4_–Ram NPs (Scheme 1). To optimize the MnFe_2_O_4_ input, PFP–HMME@PLGA/MnFe_2_O_4_–Ram NPs with different initial volumes of MnFe_2_O_4_ (40, 80, 160, and 320 µL) were prepared. Based on the concentration curves of HMME and MnFe_2_O_4_ (Figures S6 and S7, Supporting Information), the encapsulation efficiency (EE) and loading capacity (LC) of MnFe_2_O_4_ increased from 25.85 ± 2.05% to 93 ± 3.06% and from 0.16 ± 0.02% to 4.36 ± 0.25%, respectively. Meanwhile, the EE and LC of HMME increased from 56.73 ± 5.75% to 83.56 ± 2.47% and from 2.21 ± 0.21% to 3.18 ± 0.10%; subsequently, they significantly decreased to 42.73 ± 2.26% and 1.57 ± 0.21% (Figure S8, Supporting Information). An initial MnFe_2_O_4_ input of 160 µL was selected for further experiments to balance the imaging capability and sonodynamic efficacy of the NPs.

**Figure 1:**
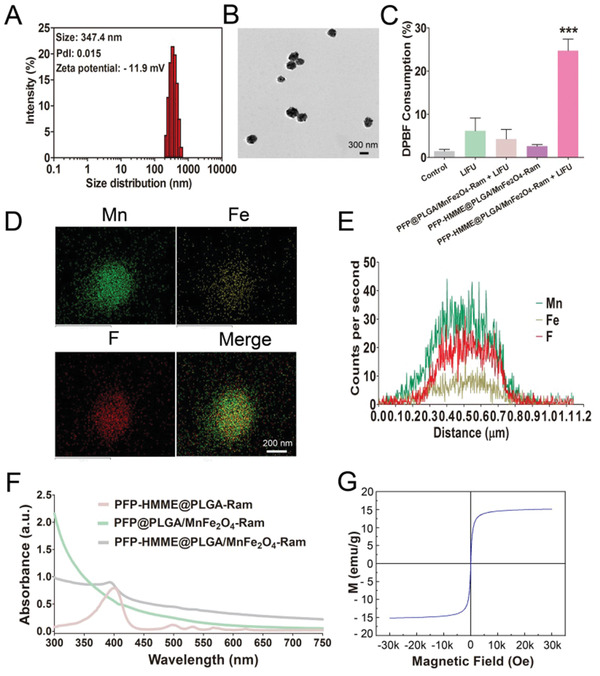
Characterization of PFP–HMME@PLGA/MnFe_2_O_4_–Ram NPs. A) The size distribution and zeta potential of NPs, as determined using DLS. B) TEM image of the NPs. C) The mixture of DPBF (50 × 10^−6^ m) and NPs (125 µg mL^−1^) was irradiated by LIFU (1.5 W cm^−2^, 1 MHz) for 150 s. Subsequently, the DPBF consumption in the indicated groups was quantified (*n* = 4). D) High‐resolution TEM elemental mapping of Mn, Fe, and F. E) TEM elemental line‐scan of the NPs. F) UV–vis absorbance spectra of the PFP–HMME@PLGA–Ram, PFP@PLGA/MnFe_2_O_4_–Ram, and PFP–HMME@PLGA/MnFe_2_O_4_–Ram NPs. G) Magnetization hysteresis loops of the NPs at 300 K ranging from −30 to +30 kOe. Data are shown as the means ± SD. C) ANOVA with Dunnett's post‐hoc test. ^***^ *p* < 0.001 versus control.

PFP–HMME@PLGA/MnFe_2_O_4_–Ram (347.4 nm, −11.9 mV) and NPs without HMME, MnFe_2_O_4_, or ramucirumab were successfully obtained (**Figure** **1**A; Figure S9, Supporting Information). Importantly, the average diameters and zeta potentials of PFP–HMME@PLGA/MnFe_2_O_4_–Ram NPs over 7 days were similar, which indicated the desirable stability and good dispersibility of the NPs (Figures S10–S13, Supporting Information). Transmission electron microscopy (TEM) images of the PFP–HMME@PLGA/MnFe_2_O_4_–Ram NPs showed a spherical morphology, where dark MnFe_2_O_4_ particles were incorporated into the PLGA shells (Figure 1B). Previously, we found that ultrasound sonochemically activated protoporphyrin IX by using hydroxyl radicals.^[^ ^21^ ^]^ As shown in Figure 1C and Figures S14 and S15 in the Supporting Information, when using 1,3‐diphenylisobenzofuran (DPBF) and singlet oxygen sensor green (SOSG) as O_2_^−^ and ^1^O_2_ sensors, respectively, PFP–HMME@ PLGA/MnFe_2_O_4_–Ram NPs plus LIFU exhibited obviously higher reactive oxygen species (ROS) production efficacy, indicating their potential as nanosonosensitizers for SDT. Additionally, elemental line‐scan mapping across the NPs revealed that Mn, Fe, and F were present in this nanoplatform (Figure 1D,E). The absorption wavelength of the PFP–HMME@PLGA/MnFe_2_O_4_–Ram NPs covered a wide range in the UV–vis–NIR region (Figure 1F; Figure S16, Supporting Information), indicating that they could be considered as candidate PA contrast agents. The paramagnetic characteristics of PFP–HMME@PLGA/MnFe_2_O_4_–Ram NPs indicated not only the successful loading of MnFe_2_O_4_, but also the potential of NPs to be used as MRI contrast agents (Figure 1G). Meanwhile, upon evaluating the LIFU‐stimulated phase‐transition property of PFP–HMME@ PLGA/MnFe_2_O_4_–Ram NPs, as shown in Figure S17 in the Supporting Information, the number of microbubbles increased and peaked at 4 min and subsequently decreased significantly after 5 min. This phenomenon indicates that the PFP–HMME@PLGA/MnFe_2_O_4_–Ram NPs could be converted into microbubbles triggered by LIFU irradiation and that it can be used as a US contrast agent, and the declining microbubbles at 5 min may be attributed to the instability of microbubbles when their volume expanded to a certain extent. Collectively, these results provide strong evidence for the successful fabrication of stable PFP–HMME@PLGA/MnFe_2_O_4_–Ram NPs and suggest that theranostic NPs are suitable for further study.

### 2.2 In Vitro RAEC‐Targeting Behavior of PFP–HMME@PLGA/MnFe_2_O_4_–Ram

**Figure 2:**
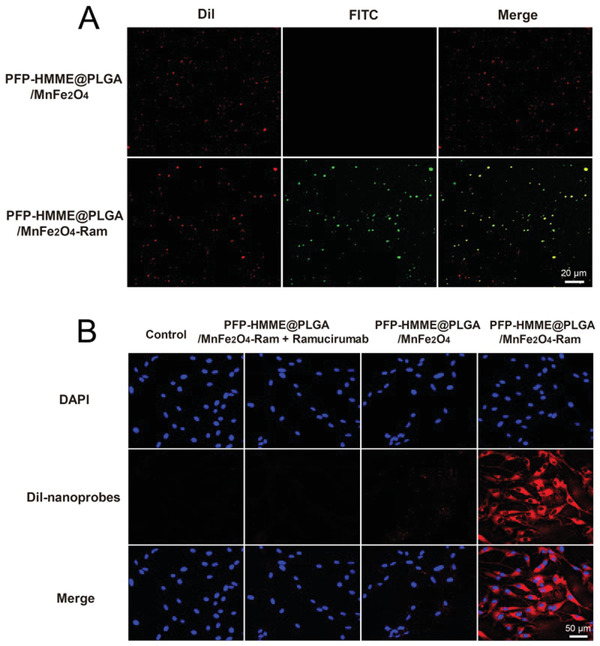
Cellular targeting behavior of PFP–HMME@PLGA/MnFe_2_O_4_–Ram NPs. A) Confocal images of DiI‐labeled NPs (red fluorescence) and FITC‐labeled PLGA–PEG–ramucirumab (green fluorescence). In merged images, the overlap of the NPs and ramucirumab appears as yellow fluorescence. B) After rabbit aortic endothelial cells (RAECs) were incubated with DiI‐labeled NPs for 2 h, the intracellular uptake of NPs in the indicated groups was observed using confocal microscopy.

The effective active targeting behavior of the PFP–HMME@PLGA/MnFe_2_O_4_–Ram nanoplatform is crucial for precise plaque angiogenesis theranostics. As shown in **Figure** **2**A, yellow fluorescence was detected by confocal laser scanning microscope (CLSM) owing to the overlap of DiI‐labeled red fluorescence and FITC‐labeled PLGA–PEG–ramucirumab green fluorescence, indicating that ramucirumab was successfully connected to the surface of NPs.

When selecting the appropriate incubation concentration and time, as shown in Figure S18 in the Supporting Information, we determined that 31.25 or 62.5 µg mL^−1^ PFP–HMME@PLGA/MnFe_2_O_4_–Ram NPs incubated with RAECs for 3–12 h was safe. Nevertheless, we selected 31.25 µg mL^−1^ NPs for subsequent experiments to reduce the influence of NPs on the viability of RAECs.

When DiI‐labeled PFP–HMME@PLGA/MnFe_2_O_4_–Ram and PFP–HMME@PLGA/MnFe_2_O_4_ NPs were incubated with RAECs for 0, 2, 4, and 6 h, CLSM and flow cytometry (FCM) demonstrated that the targeted group exhibited an increasing amount of red fluorescence in RAECs compared with the nontargeted group (Figures S19 and S20, Supporting Information). To further assess the role of ramucirumab on the targeting ability of NP shells, the VEGFR‐2 antigen was blocked by treating RAECs with free ramucirumab in advance, followed by incubation of the cells with targeted PFP–HMME@PLGA/MnFe_2_O_4_–Ram NPs for 2 h. The RAECs exhibited minimal fluorescence signals, which were similar to the results of the cells in the control and nontargeted groups (Figure 2B). This result demonstrated that ramucirumab can endow PFP–HMME@PLGA/MnFe_2_O_4_–Ram NPs with cell‐targeting ability, allowing them to be taken up by RAECs via active antigen–antibody interactions.

### 2.3 PFP–HMME@PLGA/MnFe_2_O_4_–Ram NP‐Mediated SDT Promotes RAEC Mitochondrial‐Caspase Apoptosis

**Figure 3:**
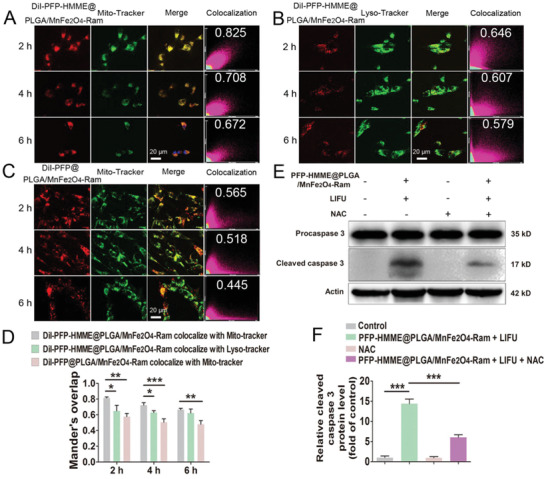
PFP–HMME@PLGA/MnFe_2_O_4_–Ram NP‐mediated SDT promotes rabbit aortic endothelial cell (RAEC) mitochondrial‐caspase apoptosis. A–D) Mitochondrial targeting behavior of PFP–HMME@PLGA/MnFe_2_O_4_–Ram NPs. After RAECs were incubated with DiI‐labeled NPs (31.25 µg mL^−1^) for 2, 4, and 6 h, the PFP–HMME@PLGA/MnFe_2_O_4_–Ram NPs colocalized with A) mitochondrial and B) lysosome trackers, and the PFP@PLGA/MnFe_2_O_4_–Ram NPs colocalized with C) mitochondrial tracker were observed and D) Mander's overlap was quantified by confocal microscopy (*n* = 5). E,F) PFP–HMME@PLGA/MnFe_2_O_4_–Ram NP‐mediated SDT induces RAEC apoptosis. RAECs were pretreated with NAC for 1 h, followed by PFP–HMME@PLGA/MnFe_2_O_4_–Ram NP‐mediated SDT. E) Representative western blot of RAECs immunoblotted and probed with antibodies against procaspase 3 and cleaved caspase 3 proteins. F) Cleaved caspase 3 protein expression relative to that of actin was quantified by densitometry (*n* = 3). Data are shown as the means ± SD. D,F) ANOVA with Tukey's post‐hoc test. ^*^ *p* < 0.05, ^**^ *p* < 0.01, and ^***^ *p* < 0.001.

The subcellular localization of PFP–HMME@PLGA/MnFe_2_O_4_–Ram NPs (31.25 µg mL^−1^) in RAECs was further evaluated. After 2, 4, and 6 h of incubation, the red fluorescence of DiI‐labeled NPs overlapped well with the green fluorescence of Mito‐tracker. Comparatively, a poor overlap was observed in the case of LysoTracker. Moreover, the mitochondrial binding ability of PFP@PLGA/MnFe_2_O_4_–Ram NPs dramatically decreased (**Figure** **3**A–D; Figure S21, Supporting Information). The 18 kDa mitochondrial translocator protein (TSPO), localized to the outer mitochondrial membrane, was confirmed to possess a high‐affinity recognition site for porphyrins.^[^ ^22^ ^]^ ALA‐PpIX selectively accumulates in the mitochondrial TSPO protein and subsequently produces ROS, releases cytochrome c, and induces macrophage mitochondrial‐caspase apoptosis upon ultrasound stimulation.^[^ ^23^ ^]^ Thus, the mitochondria‐targeting ability of PFP–HMME@PLGA/MnFe_2_O_4_–Ram NPs may be due to the specific binding of HMME to the TSPO protein.

To detect and quantify ROS production in RAECs, we determined the dichlorodihydrofluorescein (DCF) fluorescence signal using CLSM and FCM. As shown in Figures S22 and S23 in the Supporting Information, the relative fluorescence intensity in the PFP–HMME@PLGA/MnFe_2_O_4_–Ram NPs (31.25 µg mL^−1^) plus LIFU group was significantly higher than that in any other group; this fluorescence was inhibited by NAC pretreatment. Furthermore, the protein level of cleaved caspase 3 increased by 13.37‐fold in the PFP–HMME@PLGA/MnFe_2_O_4_–Ram NPs plus LIFU group compared with that in the control group; this increase was also inhibited by NAC pretreatment (Figure 3E,F). These results indicate that PFP–HMME@ PLGA/MnFe_2_O_4_–Ram NP‐mediated SDT induced mitochondrial‐caspase apoptosis via ROS production in RAECs.

### 2.4 Biocompatibility Evaluation

To assess the in vivo biosafety of PFP–HMME@PLGA/MnFe_2_O_4_–Ram NPs, blood examination and hematoxylin and eosin (H&E) staining of the main organs (heart, liver, spleen, lung, and kidney) were performed after intravenous injection of NPs on day 0, day 1, day 7, and day 28 in healthy rabbits. No obvious changes were observed in the blood indexes or histopathological lesions in the organs (Figures S24 and S25, Supporting Information). A quantitative analysis in a previous report showed that 30.33% of the 3 nm MnFe_2_O_4_ was excreted from the body of the SD rats via the renal clearance pathway, and ≈67.40% was excreted from the body via the hepatobiliary pathway within 60 h.^[^ ^10^ ^]^ The rapid excretion of MnFe_2_O_4_ within 60 h is beneficial to minimize systematic toxicity. Collectively, these results suggest that PFP–HMME@PLGA/MnFe_2_O_4_–Ram NPs exhibit high histocompatibility and are suitable for biomedical applications.

### 2.5 In Vivo Neovessel‐Targeting Behavior of PFP–HMME@PLGA/MnFe_2_O_4_–Ram

**Figure 4:**
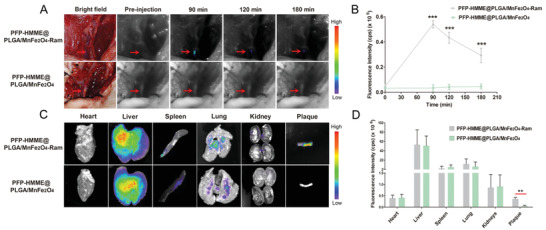
In vivo neovessel‐targeting behavior of PFP–HMME@PLGA/MnFe_2_O_4_–Ram NPs. A) The bright‐field and near‐infrared fluorescence images of femoral plaque‐bearing rabbits after intravenous injection of targeted NPs and nontargeted NPs at different time points. The red arrow in each image represents the plaque. B) The changes in fluorescence signal intensities within plaque regions at the corresponding time points (*n* = 3). C) Ex vivo fluorescence images of major organs and plaque dissected from rabbits 90 min postinjection of targeted NPs and nontargeted NPs. D) Quantitative biodistribution of targeted NPs and nontargeted NPs in rabbits, as determined by the fluorescence signal intensities of organs and plaques (*n* = 3). Data are shown as the means ± SD. B) Two‐way ANOVA with repeated measures with Tukey's post‐hoc test. D) Student's unpaired *t*‐test. ^***^ *p* < 0.001 versus 90, 120, or 180 min in the PFP–HMME@PLGA/MnFe_2_O_4_ NP group; ^**^ *p* < 0.01.

To precisely verify the biodistribution of the PFP–HMME@PLGA/MnFe_2_O_4_–Ram NPs and provide guidance for in vivo MRI/PA/US imaging and further plaque neovascularization therapy, we evaluated the living fluorescence of the plaque‐bearing rabbits in the targeted and nontargeted groups through intravenous administration of PFP–HMME@PLGA/MnFe_2_O_4_–Ram NPs and PFP–HMME@PLGA/MnFe_2_O_4_ NPs, respectively. 90 min after injection, the fluorescence signal peaked at the plaque site in the targeted group, and the fluorescence intensity in the targeted group was 16‐fold higher than that in the nontargeted group (**Figure** **4**A,B). Subsequently, the major organs and plaques were harvested 90 min after injection to evaluate the ex vivo distribution of PFP–HMME@PLGA/MnFe_2_O_4_–Ram NPs. The fluorescence signal of the excised plaque was significantly stronger in the targeted group than in the nontargeted group (Figure 4C,D). Furthermore, immunofluorescence staining showed that the DiI‐labeled targeted NPs colocalized well with CD31‐positive neovessels (Figure S26, Supporting Information). Additionally, the circulation half‐lives of PFP–HMME@PLGA/MnFe_2_O_4_ (Figure S27A, Supporting Information) and PFP–HMME@PLGA/MnFe_2_O_4_–Ram NPs (Figure S27B, Supporting Information) were calculated to be 2.48 and 7.95 h, respectively. The presumed reason for this result is that the nontargeted NPs could not specifically target plaque neovascularization and that most of them were retained in the blood circulation and prone to be rapidly eliminated by the reticuloendothelial system. Thus, these results strongly demonstrated that ramucirumab was able to endow the nanoplatform with an excellent active targeting performance, exhibiting great potential for achieving a precise multimodal imaging strategy and considerably enhanced plaque neovessel inhibition efficacy, compared to the platform developed in our previous work.^[^ ^16^ ^]^

### 2.6 In Vitro and In Vivo MRI

**Figure 5:**
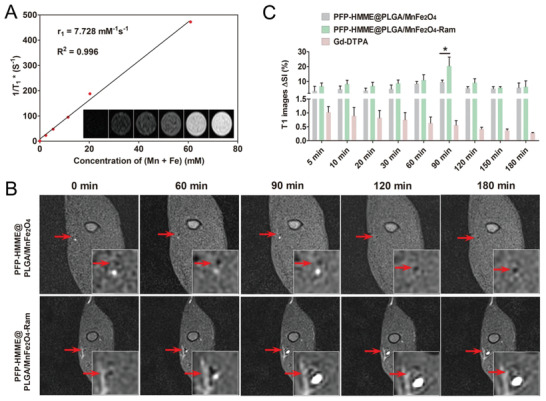
In vitro and in vivo MRI. A) In vitro MRI T1 contrast images and *R*_1_* value of PFP–HMME@PLGA/MnFe_2_O_4_–Ram dissolved in PBS at different Mn + Fe concentrations (0.014 × 10^−3^, 2.432 × 10^−3^, 5.400 × 10^−3^, 11.415 × 10^−3^, 20.260 × 10^−3^, and 60.917 × 10^−3^ m). B) In vivo MRI T1 images of rabbit femoral plaque after injection of PFP–HMME@PLGA/MnFe_2_O_4_ and PFP–HMME@PLGA/MnFe_2_O_4_–Ram NPs, as recorded at different time points. The red arrow in each transverse image represents the femoral plaque. C) Changes in MRI T1 signal intensities within plaque regions at the corresponding time points in the indicated groups (*n* = 3). Data are shown as the means ± SD. C) Two‐way ANOVA with repeated measures with Tukey's post‐hoc test. ^*^ *p* < 0.05.

MRI is a noninvasive imaging technology owing to its 3D structure and excellent soft tissue resolution.^[^ ^24^ ^]^ Here, MRI T1 imaging of PFP–HMME@PLGA/MnFe_2_O_4_–Ram NPs was systematically investigated. As shown in **Figure** **5**A, the brightness of transverse MRI contrast images of PFP–HMME@PLGA/MnFe_2_O_4_–Ram filled in Eppendorf (EP) tubes increased with an increase in the input volume of MnFe_2_O_4_. The relaxation rate *r*_1_ and *r*_2_ of the PFP–HMME@PLGA/MnFe_2_O_4_–Ram NPs, which correspond to the slope of the fitted line in Figure 5A and Figure S28 in the Supporting Information, were calculated to be 7.728 and 16.4 mm ^−1^ s^−1^ (*r*_2_/*r*_1_ = 2.12) at increasing Mn + Fe concentrations from 0.014 × 10^−3^ to 60.917 × 10^−3^ m. This rate confirmed the outstanding MRI T1 imaging properties of the PFP–HMME@PLGA/MnFe_2_O_4_–Ram NPs.

Based on the desirable in vitro MRI performance of PFP–HMME@PLGA/MnFe_2_O_4_–Ram, the in vivo contrast‐enhanced MRI performance was further assessed in plaque‐bearing rabbits. After intravenous injection of PFP–HMME@PLGA/MnFe_2_O_4_–Ram NPs (1 mg mL^−1^, 50 mL, Fe + Mn: 269.7 mg L^−1^), an obvious bright effect was observed in the plaque region, where the signal peaked at 90 min postinjection. However, the plaque‐enhanced signal of PFP–HMME@PLGA/MnFe_2_O_4_ did not change. In contrast to the results from the living fluorescence imaging study (Figure 4A,B), the enhanced signal intensity (SI) of the targeted NP group was significantly higher than that of the nontargeted group only 90 min after injection. There may be two reasons for this phenomenon: one is that immature and tortuous neovessels nonspecifically entrap the NPs, resulting in the enhancement of MRI signal of nontargeted NPs. On the other hand, the relatively low sensitivity of MRI cannot distinguish the slight difference in signal between the targeted NPs and nontargeted NPs (Figure 5B,C). Notably, after injection of Magnevist (Gd‐DTPA, Gd: 269.4 mg L^−1^), the enhanced signal in the plaque area was significantly lower than that in the targeted and nontargeted groups, and it dramatically decreased with time. According to the in vitro results of this study, the *r*_1_ value of PFP–HMME@PLGA/MnFe_2_O_4_–Ram NP was approximately twice that of Omniscan (Gd‐DTPA‐BMA). Therefore, MnFe_2_O_4_‐loaded targeted NPs exhibited not only stronger MRI T1 imaging effects but also longer aggregation time in the plaque compared with the Gd reagent, which is an appropriate MRI contrast agent.

### 2.7 In Vitro and In Vivo PA Imaging

**Figure 6:**
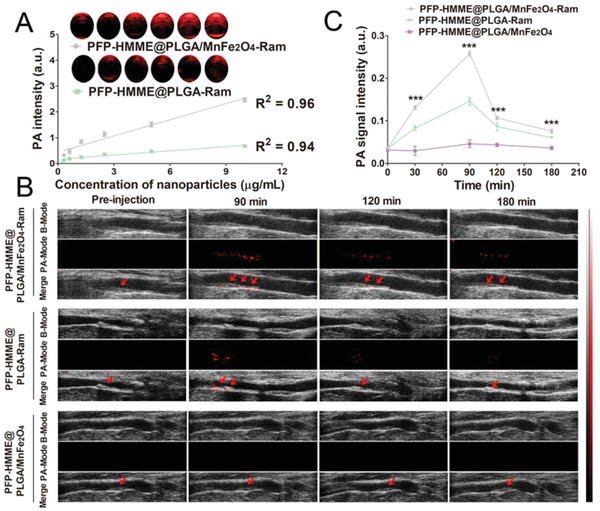
In vitro and in vivo PA imaging. A) In vitro PA contrast images and PA values of PFP–HMME@PLGA/MnFe_2_O_4_–Ram and PFP–HMME@PLGA–Ram NPs at different concentrations (0.3125, 0.625, 1.25, 2.5, 5, and 10 µg mL^−1^) (*n* = 3). B) In vivo PA images and C) signal intensities of the femoral plaques in the rabbits after injection of NPs at different time points in the indicated groups (*n* = 3). Data are shown as the means ± SD. C) Two‐way ANOVA with repeated measures with Tukey's post‐hoc test. ^***^ *p* < 0.001 versus 30, 90, 120, or 180 min in the PFP–HMME@PLGA–Ram and PFP–HMME@PLGA/MnFe_2_O_4_ NP groups.

PA imaging is a new imaging technology that can compensate for the relatively low sensitivity of MRI.^[^ ^25^ ^]^ Additionally, after injection of a contrast agent, PA imaging technology can effectively distinguish between neovessels and maturing microvasculature.^[^ ^26^ ^]^ HMME‐encapsulated PLGA NPs have previously been used as PA contrast agents.^[^ ^27^ ^]^ After full spectrum scanning from 680 to 950 nm (interval = 5 nm) in PA imaging, we observed that the wavelengths of 680 and 690 nm were the optimal wavelengths for PFP–HMME@ PLGA–Ram and PFP–HMME@ PLGA/MnFe_2_O_4_–Ram NP‐enhanced PA imaging, respectively (Figures S29 and S30, Supporting Information). Therefore, the PA imaging performances of the PFP–HMME@PLGA–Ram and PFP–HMME@PLGA/MnFe_2_O_4_–Ram NPs were assessed in vitro at 680 and 690 nm. As depicted in **Figure** **6**A, the PA signals of the PFP–HMME@PLGA–Ram and PFP–HMME@PLGA/MnFe_2_O_4_–Ram NPs increased with increasing NP concentration. More importantly, the PA signal of PFP–HMME@ PLGA/MnFe_2_O_4_–Ram was much higher than that of the PFP–HMME@PLGA–Ram NPs. This result suggests that the addition of MnFe_2_O_4_ can significantly improve the PA imaging effect of PFP–HMME@PLGA/MnFe_2_O_4_–Ram NPs.

The enhanced PA imaging effect of PFP–HMME@PLGA/MnFe_2_O_4_–Ram NPs is valuable for earlier and more accurate assessment of plaque neovascularization. After injection of PFP–HMME@PLGA/MnFe_2_O_4_–Ram, PFP–HMME@PLGA–Ram, and PFP–HMME@PLGA/MnFe_2_O_4_ NPs into plaque‐bearing rabbits, the PA signal within the plaque region was recorded at 0, 30, 90, 120, and 180 min after injection. Before injection, there were no obvious differences in the PA signals among the three groups. However, the signal of the plaque in the PFP–HMME@PLGA/MnFe_2_O_4_–Ram NP group started to increase after injection and reached a peak at 90 min. Consistent with the in vitro study, the PA signal of the plaque in the PFP–HMME@PLGA/MnFe_2_O_4_–Ram group was much higher than that in the PFP–HMME@PLGA–Ram and PFP–HMME@PLGA/MnFe_2_O_4_ NPs group (Figure 6B,C). The remarkable PA signal detected in the PFP–HMME@PLGA/MnFe_2_O_4_–Ram NP group was primarily based on the addition of MnFe_2_O_4_ and the excellent active targeting behavior of the NPs for plaque neovascularization, demonstrating the desirable performance of PFP–HMME@PLGA/MnFe_2_O_4_–Ram NPs as PA imaging contrast agents.

### 2.8 In Vitro and In Vivo US Imaging

**Figure 7:**
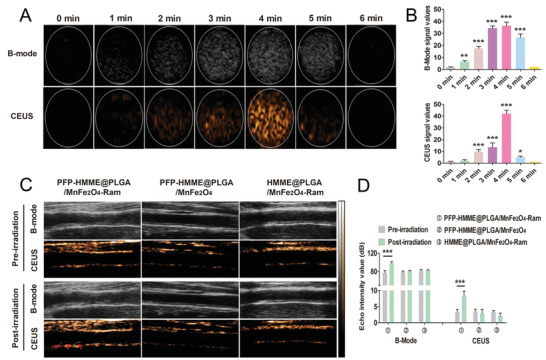
In vitro and in vivo US imaging. A) B‐mode and CEUS images and B) echo intensity values of PFP–HMME@PLGA/MnFe_2_O_4_–Ram NPs irradiated using LIFU (1.5 W cm^−2^, 1 MHz) for different durations (*n* = 5). C) US images in B‐mode and CEUS of rabbit femoral plaques 90 min after intravenous injection with PFP–HMME@PLGA/MnFe_2_O_4_–Ram NPs, PFP–HMME@PLGA/MnFe_2_O_4_ NPs, and HMME@PLGA/MnFe_2_O_4_–Ram NPs pre‐LIFU irradiation and postirradiation (1.5 W cm^−2^, 1 MHz, 15 min). The red arrows indicate the microbubbles in the plaque. D) Echo intensity values in B‐mode and CEUS of the three groups before and after LIFU irradiation (*n* = 5). Data are shown as the means ± SD. B) ANOVA with Dunnett's post‐hoc test. D) Student's paired *t*‐test. ^*^ *p* < 0.05, ^**^ *p* < 0.01, and ^***^ *p* < 0.001 versus 0 min; ^***^ *p* < 0.001.

Ultrasound is a real‐time, noninvasive, and deep‐penetrating imaging technology that is complementary to MRI and PA imaging. Within 0–6 min of LIFU irradiation in vitro, the echo intensity in both B‐mode and contrast‐enhanced ultrasound (CEUS) from the PFP–HMME@PLGA/MnFe_2_O_4_–Ram NPs gradually increased and peaked at 4 min and, subsequently, significantly decreased after 5 min (**Figure** **7**A,B), consistent with the microscopy results for the ADV of the PFP–HMME@PLGA/MnFe_2_O_4_–Ram NPs (Figure S17, Supporting Information).

Based on the time of maximal plaque accumulation of the PFP–HMME@PLGA/MnFe_2_O_4_–Ram NPs guided by MRI and PA imaging, in vivo US imaging was conducted on the plaque‐bearing rabbits. At 90 min after injection, compared with preirradiation, the US echo signal enhancement in the PFP–HMME@PLGA/MnFe_2_O_4_–Ram NP group significantly increased after LIFU irradiation for 15 min. However, the echo intensity in the PFP–HMME@ PLGA/MnFe_2_O_4_ NPs and HMME@PLGA/MnFe_2_O_4_–Ram NP groups did not change before or after LIFU irradiation (Figure 7C,D), which suggests that the PFP encapsulated in the NPs could generate microbubbles in situ during LIFU irradiation, allowing them to act as US contrast imaging agents.

Collectively, the above results indicate that the PFP–HMME@PLGA/MnFe_2_O_4_–Ram NPs hold promise as effective atherosclerotic plaque neovessel accumulation and multimodal imaging agents. The integration of MRI, PA, and US into a single nanoplatform could facilitate real‐time observation of the distribution of the nanosonosensitizer for adjustment of therapeutic time windows.

### 2.9 PFP–HMME@PLGA/MnFe_2_O_4_–Ram NP‐Mediated SDT Suppresses Neovascularization and Increases the Stability of Advanced Atherosclerotic Plaque in Rabbits

Pathological angiogenesis acts as a conduit for the entry of red blood cells, lipids, and macrophages into atherosclerotic plaques.^[^ ^28^ ^]^ Hemoglobin ingestion by macrophages activates the expression of hypoxia inducible factor‐1*α* (HIF‐1*α*), which is a consequence of intracellular Fe^2+^ deprivation and prolyl hydroxylase 2 (PHD2) inhibition. VEGF‐A, MMP‐2, and MMP‐9, which are secreted by macrophages via HIF‐1*α* activation, further promote extracellular collagen degradation and abnormal proliferation of angiogenesis.^[^ ^16^, ^29^ ^]^ Therefore, effective inhibition of plaque neovascularization is of great significance for reducing intraplaque hemorrhage and inflammation, subsequently stabilizing the plaque.

**Figure 8:**
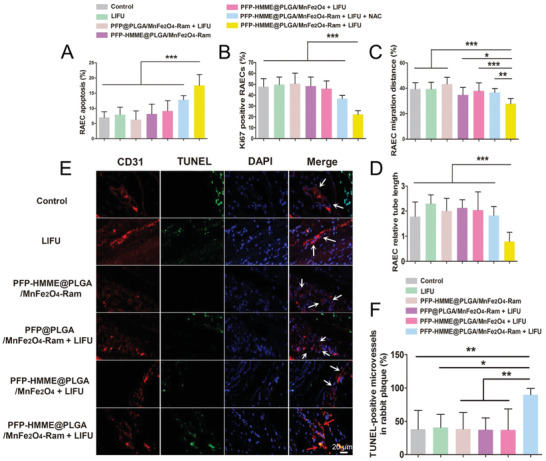
PFP–HMME@PLGA/MnFe_2_O_4_–Ram‐mediated SDT induces apoptosis of the neovessels in the rabbit femoral atherosclerotic plaque. A–D) PFP–HMME@PLGA/MnFe_2_O_4_–Ram‐mediated SDT promoted rabbit aortic endothelial cells (RAECs) apoptosis and inhibited proliferation, migration, and tube formation of RAECs. RAECs were pretreated with NAC for 1 h, followed by SDT treatment. Quantification of RAEC A) apoptosis, B) proliferation, C) migration 6 h after scratching, and D) tube formation (*n* = 4, 5, 4, and 4, respectively). Data were obtained from three independent experiments. E,F) PFP–HMME@PLGA/MnFe_2_O_4_–Ram‐mediated SDT induces neovascular endothelial cell apoptosis on day 3 after treatment of the advanced plaque in the rabbits. E) Representative sections and F) quantification of the percentage of TUNEL‐positive microvessels in the plaque (*n* = 5). White and red arrows indicate TUNEL‐negative and TUNEL‐positive microvessels, respectively. Data are shown as the means ± SD. A–D,F) ANOVA with Dunnett's post‐hoc test. ^*^ *p* < 0.05, ^**^ *p* < 0.01, and ^***^ *p* < 0.001.

**Figure 9:**
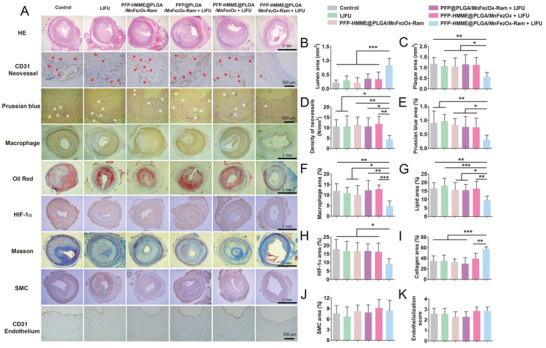
PFP–HMME@PLGA/MnFe_2_O_4_–Ram‐mediated SDT inhibits plaque neovascularization and stabilizes atherosclerotic plaque on day 28 after treatment of the advanced plaque in rabbits. A) Representative histopathological staining of plaque sections and B–K) quantification (*n* = 7). Red arrows indicate abnormal adventitial neovessels. White arrows indicate intraplaque hemorrhage. Data are shown as the means ± SD. B–K) ANOVA with Dunnett's post‐hoc test. ^*^ *p* < 0.05, ^**^ *p* < 0.01, and ^***^ *p* < 0.001.

In this study, after incubating PFP–HMME@PLGA/MnFe_2_O_4_–Ram NPs with RAECs for 2 h, PFP–HMME@PLGA/MnFe_2_O_4_–Ram NP‐mediated SDT significantly induced RAEC apoptosis and inhibited the proliferation, migration, and tubulogenesis of RAECs (**Figure** **8**A–D). In the femoral artery region of the normal rabbit, we found that the highest local temperature was 35.8 °C within 15 min of LIFU irradiation. Moreover, the temperature change was no more than 1 °C throughout the treatment (Figure S31, Supporting Information). Additionally, 90 min after intravenous injection of PFP–HMME@PLGA/MnFe_2_O_4_–Ram NPs, PFP–HMME@PLGA/MnFe_2_O_4_–Ram NP‐mediated SDT was performed in the plaques of the rabbits. On day 3 after treatment, the number of apoptotic neovessels increased ≈1.35‐fold (Figure 8E,F), and the hypoxic area was reduced by ≈50% compared with that in the control group (Figure S32, Supporting Information). On day 28 after treatment, compared with the corresponding values in the control group, the lumen area and plaque area significantly increased by 272.3% and decreased by 51.8%, respectively (**Figure** **9**A–C). More importantly, the density of neovessels in the group subjected to PFP–HMME@PLGA/MnFe_2_O_4_–Ram NP‐mediated SDT decreased by 59.1% (Figure 9D), while that in the DVDMS‐SDT group decreased by 52.9%,^[^ ^16^ ^]^ suggesting that targeted NP‐mediated SDT was better than DVDMS‐SDT in terms of inhibition of plaque neovascularization. Furthermore, PFP–HMME@PLGA/MnFe_2_O_4_–Ram NP‐mediated SDT significantly decreased the percentage of intraplaque hemorrhage by 66.2% (Figure 9E). A previous study confirmed that blockade of VEGFR‐2 inhibits intraplaque hemorrhage by normalizing plaque neovessels.^[^ ^30^ ^]^ However, compared with the control group, there was no reduction in intraplaque hemorrhage in the PFP–HMME@PLGA/MnFe_2_O_4_–Ram group. Thus, the reduction of intraplaque hemorrhage is due to the inhibition of plaque neovascularization by the targeted NP‐mediated SDT, rather than the maturation of neovessels caused by ramucirumab. The targeted NP‐mediated SDT reduced the macrophage content by 59%, lipids by 41.9%, and HIF‐1*α* by 48%, and increased collagen content by 67.4% compared with the control group (Figure 9F–I). PET‐computed tomography (CT) imaging showed that SDT obviously reduced the inflammation level in the femoral plaque by 71.5% compared with the control group (Figure S33, Supporting Information). The decrease in inflammatory cells and increase in collagen content are important indicators of plaque stability. At day 28 after treatment, SDT did not change the smooth muscle cell (SMC) content in the plaque (Figure 9J), suggesting that SDT has no impact on collagen synthesis. Macrophage foam cells are the main inflammatory cells in the plaque and can express MMP‐2 and MMP‐9 to degrade collagen.^[^ ^31^ ^]^ After inhibition of neovessels by SDT, the number of macrophages entering the plaque through neovessels decreased significantly, and the expression and secretion of MMP‐2 and MMP‐9 also decreased, resulting in the reduction of collagen degradation. Notably, SDT had no effect on the coverage of endothelial cells in the lumen side (Figure 9K), indicating that targeted NP‐mediated SDT can safely and effectively inhibit intraplaque hemorrhage and inflammation and finally stabilize the plaque via the inhibition of neovascularization.

### 2.10 PFP–HMME@PLGA/MnFe_2_O_4_–Ram NP‐Mediated SDT Is Safe for Plaque‐Bearing Rabbits

**Figure 10:**
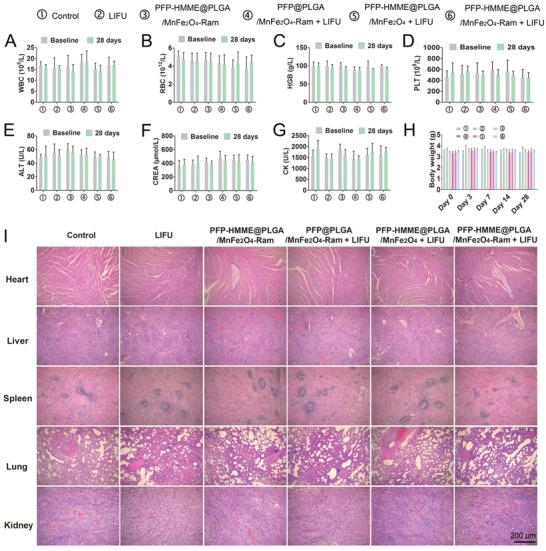
In vivo biocompatibility evaluations. A–G) Blood examination of the plaque‐bearing rabbits at baseline and day 28 following treatment in the indicated groups (*n* = 6). H) Time‐dependent body weight changes of the plaque‐bearing rabbits in the indicated groups (*n* = 5). I) Representative H&E staining of the plaque‐bearing rabbit organs (heart, liver, spleen, lung, and kidney) excised at day 28 after treatment in the indicated groups. Data are shown as the means ± SD. A–H) Two‐way ANOVA with repeated measures with Tukey's post‐hoc test.

The potential in vivo toxicity of PFP–HMME@PLGA/MnFe_2_O_4_–Ram NP‐mediated SDT was further evaluated in plaque‐bearing rabbits. On day 28 after PFP–HMME@PLGA/MnFe_2_O_4_–Ram NP‐mediated SDT, the changes in blood biochemical indices and weight fluctuations were negligible compared with the corresponding baseline values (**Figure** **10**A–H). Moreover, histopathological changes, according to H&E staining of the major organs, indicated undetectable toxicity in rabbits compared with the other groups (Figure 10I).

In order to assess the effect of SDT on endothelial cell function, we previously found that the limb perfusion and resistance index downstream of the atherosclerotic lesion in the femoral artery were similar before and after SDT.^[^ ^23^ ^]^ In this study, 28 days after PFP–HMME@PLGA/MnFe_2_O_4_–Ram NP‐mediated SDT, flow‐mediated vasodilation (FMD%), as a marker for endothelial cell function assessed by ultrasonography, did not change significantly compared with baseline (Figure S34, Supporting Information). Moreover, the expression of VCAM‐1 by endothelial cells in the femoral atherosclerotic plaque was not different between the control and PFP–HMME@PLGA/MnFe_2_O_4_–Ram NP‐mediated SDT group (Figure S35, Supporting Information). These results demonstrate the high therapeutic biosafety of FP‐HMME@PLGA/MnFe_2_O_4_–Ram NP‐mediated SDT in combating plaque neovascularization.

## 3 Conclusion

In this study, a safe, LIFU‐responsive, mitochondria‐targeting, and multifunctional theranostic nanoplatform, PFP–HMME@PLGA/MnFe_2_O_4_–Ram, was successfully fabricated by encapsulating MnFe_2_O_4_, HMME, and PFP into PLGA with ramucirumab surface modification. Based on MRI/PA/US multimodal imaging, PFP–HMME@PLGA/MnFe_2_O_4_–Ram NP‐mediated SDT inhibited plaque neovascularization by inducing mitochondrial‐caspase apoptosis in neovascular endothelial cells without causing obvious side effects. A clinical randomized controlled trial showed that SDT rapidly reduced plaque inflammation and improved walking performance in patients with symptomatic femoropopliteal peripheral artery disease.^[^ ^32^ ^]^ The construction of a nanosonosensitizer enables real‐time monitoring of the distribution of sonosensitizers at the molecular level, which is more convenient for clinical use, and also makes SDT more accurate and efficient. In the near future, this study will provide a solid foundation for clinical randomized controlled trials of atherosclerotic plaque neovascularization theranostics.

## 4 Experimental Section

### Materials

Carboxyl‐modified PEGylated poly(lactic‐*co*‐glycolic acid) (lactide:glycolide = 50:50, PLGA = 25 000 Da MW, PEG = 5000 Da MW; PLGA–PEG_5000_–COOH) was obtained from Jinan Daigang Biomaterial Co., Ltd. (Shandong, China). Oleic acid‐coated manganese ferrite (MnFe_2_O_4_) NPs (*d* = 3 nm, 8 mg mL^−1^) were purchased from Ruixi Biotechnology Co., Ltd. (Xi'an, China). HMME was purchased from Shanghai DB Chemical Technology Co., Ltd. (Shanghai, China). DPBF was obtained from Sigma‐Aldrich Co. (Missouri, USA). SOSG was purchased from Thermo Fisher Scientific (Shanghai, China). *N*‐acetyl‐l‐cysteine (NAC) was obtained from Beyotime Biotechnology Co., Ltd. (Shanghai, China). Ramucirumab (Cyramza) was purchased from Eli Lilly Inc. (Indianapolis, IN, USA). PLGA–PEG–ramucirumab (average 289 621.5 Da MW, PLGA–PEG_5000_–COOH:ramucirumab ≈ 5:1) was fabricated by Chongqing Punuo Microbian Technology Co., Ltd. (Chongqing, China) using the carbodiimide method (Figure S5, Supporting Information). PFP, poly(vinyl alcohol) (PVA; 25 000 MW), 2,7‐dichlorodihydro fluoresce‐eindiacetate diacetate (DCFH‐DA), 1,1‐dioctadecyl‐3,3,3′,3′‐tetramethylindo carbocyanine perchlorate (DiI), 1,1′‐dioctadecyl‐3,3,3′,3′‐tetramethylindotricarbocyanine iodide (DiR), and 2‐(4‐amidinophenyl)‐6‐indolecarbamidine dihydrochloride (DAPI) were obtained from Sigma‐Aldrich Co. (Missouri, USA). All reagents used in this study were of analytical grade.

### Synthesis of PFP–HMME@PLGA/MnFe_2_O_4_–Ram NPs

PLGA–PEG–ramucirumab (50 mg) encapsulating HMME (2 mg),^[^ ^33^ ^]^ MnFe_2_O_4_ (160 µL), and PFP (200 µL) were fabricated via a simple double‐emulsion method, as previously described.^[^ ^33^ ^]^ The preparation of PFP–HMME@PLGA/MnFe_2_O_4_ NPs was fabricated as described above, except that PLGA–PEG–ramucirumab was replaced by PLGA–PEG at the same molar ratio. DiI‐ or DiR‐labeled NPs and NPs without HMME, PFP, or MnFe_2_O_4_ were fabricated using the same method.

### Characterization of PFP–HMME@PLGA/MnFe_2_O_4_–Ram NPs

The size and zeta potential of the PFP–HMME@PLGA/MnFe_2_O_4_–Ram NPs and NPs without HMME, MnFe_2_O_4_, or ramucirumab were measured using dynamic light scattering (DLS, Malvern Instruments Ltd., UK). The structure and morphology of the NPs were characterized by TEM (Hitachi H‐7600, Japan). To further verify the surface distribution of MnFe_2_O_4_, high‐resolution images, elemental mapping images, and energy‐dispersive spectroscopy (EDS) line‐scan measurements were obtained by TEM (JEM 2100, JEOL Ltd., Tokyo, Japan). The mean particle size and zeta potential of the PFP–HMME@PLGA/MnFe_2_O_4_–Ram NPs dissolved in phosphate‐buffered solution (PBS) or 5% (vol/vol) FBS RPMI‐1640 culture medium were measured over a prolonged period (1, 2, 3, 4, 5, 6, and 7 days). The UV absorption spectra of the NPs were obtained using a spectrophotometer (UV‐3600, Shimadzu, Japan). Magnetization hysteresis loops of 3 nm MnFe_2_O_4_ and PFP–HMME@PLGA/MnFe_2_O_4_–Ram NPs were obtained using a vibrating sample magnetometer (PPMS‐9, MicroSense, USA). PFP–HMME@PLGA/MnFe_2_O_4_–Ram NPs were destroyed using DMSO. The organic and aqueous phases were separated for HMME detection using a spectrophotometer. MnFe_2_O_4_ loading was determined by dissolving NPs (50 mg) in 2% nitric acid solution and measuring the amount of MnFe_2_O_4_ in the NPs using inductively coupled plasma optical emission spectrometry (ICP‐OES, PerkinElmer Optima 8000, USA). The EE and LC of HMME and MnFe_2_O_4_ in the NPs were calculated as previously described.^[^ ^34^ ^]^ To confirm the conjugation of ramucirumab onto the shell of the NPs, FITC‐labeled PLGA–PEG–ramucirumab and DiI NPs encapsulating HMME, MnFe_2_O_4_, and PFP were analyzed under a Nikon A1 CLSM (Japan).

### ROS Generation and Liquid–Gas Phase‐Change Properties of PFP–HMME@PLGA/MnFe_2_O_4_–Ram NPs by LIFU Activation

ROS production in PFP–HMME@PLGA/MnFe_2_O_4_–Ram NPs following LIFU activation was measured using DPBF and SOSG, as previously described.^[^ ^33^ ^]^ Briefly, a mixture of DPBF (50 × 10^−6^ m) and NPs (125 µg mL^−1^) was irradiated using LIFU (1.5 W cm^−2^, 1 MHz) (LM.SC051 ACA, Institute of Ultrasound Imaging of Chongqing Medical Sciences, Chongqing, China) for 0, 30, 60, 90, 120, and 150 s in the dark. Subsequently, the absorbance and consumption of DBPF in each group were determined using a UV–vis spectrophotometer. The mixture of SOSG (5 × 10^−6^ m) and NPs (31.25, 62.5, 125, and 250 µg mL^−1^) was irradiated using LIFU (1.5 W cm^−2^, 1 MHz) for 0, 30, 60, and 90 s in the dark. Next, the fluorescence intensity of SOSG in each group was determined using a fluorescence spectrophotometer. The LIFU‐stimulated liquid–gas phase transition of PFP in the PFP–HMME@PLGA/MnFe_2_O_4_–Ram NPs was investigated at a power of 1.5 W cm^−2^ over a duration of 0–6 min using an optical microscope (Olympus IX53, Canada).

### Cell Culture and Cytotoxicity Assay

RAECs were obtained from Procell Life Science&Technology Co., Ltd. (Wuhan, China). The cells were cultured in RPMI‐1640 medium supplemented with 10% FBS and 1% penicillin/streptomycin and maintained in an incubator with a humidified atmosphere containing 5% CO_2_ at 37 °C. To assess the binding ability of ramucirumab with RAECs, RAECs were seeded in 35 mm Petri dishes at a density of 5 × 10^5^ cells for 24 h. The culture medium was then replaced with fresh culture medium containing different concentrations of FITC‐labeled ramucirumab (0, 0.3125, 0.625, 1.25, 2.5, and 5 mg mL^−1^). After 3 h of incubation, the FITC‐positive RAECs were analyzed using an FCM system (FACS Vantage, BectonDickinson, USA). RAECs seeded in 96‐well culture plates at a density of 1 × 10^4^ cells per well were incubated with PFP–HMME@PLGA/MnFe_2_O_4_–Ram NPs at different concentrations (0, 31.25, 62.5, 125, 250, and 500 µg mL^−1^). After 3, 6, 12, and 24 h of incubation, the CCK‐8 assay (Kumamoto, Japan) was used to evaluate cell viability.

### Intracellular ROS Measurement

Intracellular ROS generation was determined using DCFH‐DA, as previously described.^[^ ^35^ ^]^ Briefly, seeded RAECs (2 × 10^5^ cells mL^−1^) were preincubated with NAC (5 × 10^−3^ m) for 1 h. Following co‐incubation with different NPs (100 µL, 31.25 µg mL^−1^) for 2 h, the DCFH‐DA was added into each dish and incubated for 30 min. Immediately after LIFU (1.5 W cm^−2^, 1 MHz, 90 s) activation, the cells were photographed under a fluorescence microscope. The fluorescence of DCF was assessed at 488 nm excitation and 525 nm emission wavelengths using a multimode microplate reader (Tecan Infinite M200 Pro, Mannedorf, Switzerland) and analyzed by FCM.

### Western Blotting

Western blotting was performed as previously described.^[^ ^16^ ^]^ Briefly, after gel electrophoresis, transfer, and blocking, the protein samples of RAECs were incubated with anti‐caspase 3 (1:1000, bs‐2593R) and anticleaved caspase 3 (1:1000, bs‐20363R) primary antibodies overnight at 4 °C. Antiactin (1:1000, CST, Cat#4967) was used as a loading control. Horseradish peroxidase (HRP)‐conjugated IgG secondary antibodies (1:4000) were used. Protein bands were visualized, quantified, and normalized to actin levels.

### RAEC Apoptosis, Proliferation, Migration, and Tube Formation Assay

After incubating PFP–HMME@PLGA/MnFe_2_O_4_–Ram NPs with RAECs for 2 h, the apoptosis, proliferation, migration, and tubulogenesis assays of RAECs were performed 6 h after LIFU activation as previously described.^[^ ^16^ ^]^ The apoptotic rate of RAECs was detected using Annexin V:FITC Apoptosis Detection Kits (BD Pharmingen) and analyzed using the BD FACSDiva Software v7.0 (Becton‐Dickinson, USA). 6 h after PFP–HMME@PLGA/MnFe_2_O_4_–Ram NP‐mediated SDT, the cells were fixed, blocked, and incubated overnight with anti‐Ki67 (1:200, Abcam, Cat#ab16667), followed by antirabbit secondary antibodies (1:400) for 1 h. Subsequently, the cells were counterstained with DAPI for 15 min at 37 °C in the dark. Finally, Ki67‐positive cells were visualized and counted using fluorescence microscopy. After SDT treatment, the RAECs were wounded by manual scraping with a 200 µL pipette tip. Subsequently, cell migration was photographed at the same locations. Phase‐contrast images were recorded 6 h after scratching using a microscope. The width of the wound at 4× magnification in three random fields of each well was chosen and measured using IPP software to determine the healed distance. To perform the tube formation assay, each well in a 96‐well plate was coated with 60 µL of growth factor‐reduced Matrigel (Corning, Cat#356234) and incubated at 37 °C for polymerization. After SDT treatment, the RAECs were trypsinized, resuspended in the culture medium, and seeded in the wells (1–2 × 10^4^ cells per well) for 24 h. Tube formation was observed and photographed using a microscope. At least three representative images at 4× magnification of each well were recorded and analyzed using the IPP software.

### Advanced Rabbit Atherosclerotic Plaque Models

Adult male New Zealand rabbits (2.5–3 kg) were purchased from and housed at the Animal Center of Chongqing Medical University. The rabbits were fed a 1.5% high‐cholesterol diet 2 weeks prior to the balloon denudation of the right femoral artery, followed by the same diet for another 6 weeks and a 4‐week normal diet, as previously described.^[^ ^16^ ^]^ For SDT treatment, the plaque‐bearing rabbits were sedated with intramuscular (i.m.) injection of ketamine (25 mg kg^−1^), xylazine (5 mg kg^−1^), and acepromazine (0.75 mg kg^−1^). Anesthesia was maintained using 1% isoflurane delivered in oxygen. At 90 min after intravenous administration of PFP–HMME@PLGA/MnFe_2_O_4_–Ram NPs, the anesthetized rabbits were subjected to LIFU (1.5 W cm^−2^, 1 MHz) activation for 15 min based on the previous results.^[^ ^23^ ^]^ All animal experiments were performed in accordance with the guidelines of the Animal Ethics Committee of Chongqing Medical University.

### In Vivo Thermal Effect Evaluation Caused by LIFU Irradiation

A normal rabbit was anesthetized and the region of the right femoral artery was exposed to LIFU irradiation (1.5 W cm^−2^, 1 MHz) for 15 min. Rabbits that were not subjected to LIFU irradiation were used as controls. An infrared thermal imaging camera (Fluke Ti32, USA) was used to monitor local temperature changes in the rabbit femoral artery region during ultrasound irradiation. This independent experiment was repeated three times.

### In Vitro and In Vivo Ramucirumab Targeting Studies

RAECs were incubated with DiI‐labeled PFP–HMME@PLGA/MnFe_2_O_4_–Ram or PFP–HMME@PLGA/MnFe_2_O_4_ NPs (31.25 µg mL^−1^) for 0, 2, 4, or 6 h, respectively. Subsequently, the cells were observed by CLSM after staining with DAPI and analyzed by flow cytometry. Additionally, DiI‐labeled PFP–HMME@PLGA/MnFe_2_O_4_–Ram NPs, PFP–HMME@PLGA/MnFe_2_O_4_–Ram NPs, an appropriate amount of free ramucirumab, and nontargeted PFP–HMME@PLGA/MnFe_2_O_4_ NPs were added to the RAECs. After 2 h of incubation and staining with DAPI, RAECs were observed using CLSM.

After 2, 4, or 6 h of incubation with PFP–HMME@PLGA/MnFe_2_O_4_–Ram or PFP@PLGA/MnFe_2_O_4_–Ram NPs labeled with DiI, the RAECs were incubated with 50 × 10^−9^ m MitoTracker Deep Red FM (Cat#M22426, Invitrogen) for 30 min to label the mitochondria or with 75 × 10^−9^ m LysoTracker (Cat#L7526, Invitrogen) to label the lysosomes. The cells were washed twice with PBS, and the mitochondrial/lysosomal localization of the corresponding NPs was confirmed by co‐staining with DiI, MitoTracker, or LysoTracker using CLSM. Subsequently, the Mander's overlap of each image was measured. To further assess the mitochondrial localization of the PFP–HMME@PLGA/MnFe_2_O_4_–Ram NPs, the RAECs were incubated with PFP–HMME@PLGA/MnFe_2_O_4_–Ram or PFP@PLGA/MnFe_2_O_4_–Ram NPs for 2 h. The samples were handled as previously described^[^ ^10^ ^]^ and analyzed using a Philips EM280S TEM.

The plaque‐bearing rabbits were divided into targeted and nontargeted groups and intravenously administered DiR‐labeled NPs (50 mg per rabbit). After anesthetizing with 3% pentobarbital sodium (60 mg mL^−1^), the femoral arteries were surgically exposed, and a live fluorescence imaging (Fx7 Ir Spectra, Vilber Lourmat, France) system was used to evaluate the biodistribution of NPs at different times (preinjection, 90, 120, and 180 min postinjection). The rabbits in the two groups were sacrificed by injecting an overdose of pentobarbital (160 mg kg^−1^) at 90 min postinjection, and the major organs (heart, liver, spleen, lung, and kidney) and plaques were harvested for ex vivo fluorescence imaging. In addition, the rabbits received the same treatment as described above, except that the NPs were labeled with DiI. At 90 min postinjection, the plaques were immediately dissected for frozen sections (7 µm). Subsequently, the frozen sections were fixed, blocked, and incubated with anti‐CD31 antibody (1:20, Cat#ab9498, Abcam). After overnight incubation at 4 °C, the sections were washed thrice with PBS and incubated with FITC‐conjugated antimouse IgG for 1 h at 37 °C. The nuclei were counterstained with DAPI and the sections were visualized using CLSM.

### In Vitro and In Vivo MRI

MRI experiments were conducted using a clinical 3 T MR scanner (HDXT2012; GE Medical Systems, Fairfield, USA). PFP–HMME@PLGA/MnFe_2_O_4_–Ram was dissolved in PBS at different input volumes of MnFe_2_O_4_ (0, 160, 320, 640, 1280, and 2560 µL, the concentrations of Mn + Fe: 0.014 × 10^−3^, 2.432 × 10^−3^, 5.400 × 10^−3^, 11.415 × 10^−3^, 20.260 × 10^−3^, and 60.917 × 10^−3^ m) and was placed in 15 mL EP tubes for in vitro MRI. The T1 imaging parameters were set as follows: TR, 115 ms; TE, 9.21 ms; flip angle, 30°; FOV, 245 mm; matrix, 256 × 256; slice thickness, 1 mm; and T2 imaging parameters were TR, 6000 ms; TE, 90 ms; flip angle, 30°; FOV, 247 mm; matrix, 266 × 384; slice thickness, 1 mm. The MRI T1‐signal and T2‐signal intensities within the region of interest (ROI) were measured, and the corresponding relaxation rates were calculated. For in vivo MRI, the plaque‐bearing rabbits (*n* = 3) were anesthetized and intravenously injected with PFP–HMME@PLGA/MnFe_2_O_4_–Ram, PFP–HMME@PLGA/MnFe_2_O_4_, or Gd‐DTPA saline solution. The imaging parameters were set as follows: TR, 3200 ms; TE, 80 ms; flip angle, 30°; FOV, 245 mm; matrix, 256 × 256; slice thickness, 1 mm. Subsequently, transverse MRI images were collected at different time points (0, 5, 10, 20, 30, 60, 90, 120, 150, and 180 min). The average T1 SI of the plaque region of the same slice was measured. The percentage of signal intensity increase (ΔSI) was calculated as follows: ΔSI = (SI_post_ − SI_pre_)/SI_pre_ × 100%.

### In Vitro and In Vivo PA Imaging

For in vitro PA imaging of PFP–HMME@PLGA/MnFe_2_O_4_–Ram (1.1272 mg HMME and 1.029 mg MnFe_2_O_4_) and PFP–HMME@PLGA–Ram (1.1272 mg HMME) NPs, the samples were first scanned with an excitation wavelength range of 680–970 nm on a PA imaging system (Vevo LAZR, Canada) at a PA gain of 40 dB. The tunable laser parameters used for the PA imaging system were set as follows: type, flash lamp pumped Q‐switched Nd:YAG laser with an optical parametric oscillator (OPO) and second harmonic generator; frequency, 20 Hz; wavelength, 680–970 nm; step size, 2 nm; pulse duration, 4–6 ns; peak energy, 45 mJ +/− 5 mJ (at 20 Hz); spot size: 24 mm^2^ (1 mm × 24 mm). The PA images and the relative PA signal values of the PFP–HMME@PLGA/MnFe_2_O_4_–Ram and PFP–HMME@PLGA–Ram NPs at different concentrations (0.3125, 0.625, 1.25, 2.5, 5, and 10 µg mL^−1^) were obtained at 690 and 680 nm. For in vivo PA imaging, plaque‐bearing rabbits (*n* = 3) were intravenously injected with PFP–HMME@PLGA/MnFe_2_O_4_–Ram, PFP–HMME@PLGA–Ram, and PFP–HMME@PLGA/MnFe_2_O_4_ NPs in saline (1 mg mL^−1^, 50 mL). Next, the PA images were collected at different time points (pre, 30, 90, 120, and 180 min), and the average PA signal intensity value of the plaque regions was measured.

### In Vitro and In Vivo US Imaging

A MyLab90 ultrasound diagnostic instrument (Esaote, Italy) was used for US imaging. To assess the in vitro liquid–gas phase transition of PFP, PFP–HMME@PLGA/MnFe_2_O_4_–Ram NPs (1.25 mg mL^−1^) incorporated in a gel phantom (2% w/v) were exposed to LIFU activation (1.5 W cm^−2^, 1 MHz). US images of the samples were captured 0–6 min after irradiation, and the corresponding echo intensity values of the ROI were quantitatively analyzed using DFY software (Institution of Ultrasound Imaging of Chongqing Medical University, Chongqing, China). For in vivo US imaging, plaque‐bearing rabbits (*n* = 3) were intravenously injected with PFP–HMME@PLGA/MnFe_2_O_4_–Ram, PFP–HMME@PLGA/MnFe_2_O_4_, and HMME@PLGA/MnFe_2_O_4_–Ram NPs in saline (1 mg mL^−1^, 50 mL). At 90 min after injection, the plaques were irradiated using LIFU at a power of 1.5 W cm^−2^ for 15 min. Subsequently, US imaging of the plaques in the three groups was performed, and the echo intensity values in the plaque region were measured.

### Apoptosis Assay of Neovessels and Hypoxia Detection in the Rabbit Plaque

Forty‐two rabbits with advanced femoral plaques were randomly assigned to the control, LIFU, PFP–HMME@PLGA/MnFe_2_O_4_–Ram, PFP@PLGA/MnFe_2_O_4_–Ram + LIFU, PFP–HMME@PLGA/MnFe_2_O_4_ + LIFU, and PFP–HMME@PLGA/MnFe_2_O_4_–Ram + LIFU groups (*n* = 7, each). On day 3 after treatment, the rabbits were sacrificed, and the cryosections were stained with anti‐CD31 antibody and TdT‐mediated dUTP nick‐end labeling (TUNEL) using the in situ cell death detection kit POD (Roche Diagnostics, Roswell, GA, USA), in accordance with the manufacturer's protocol, followed by counterstaining with DAPI to identify the nuclei. Only TUNEL‐positive cells that colocalized with DAPI and CD31 were counted as positive. The percentage of TUNEL‐positive microvessels was determined as described previously.^[^ ^16^ ^]^ Thirty rabbits with advanced femoral plaques were randomly assigned as described above (*n* = 5 each). On day 3 after treatment, hypoxia in the plaque was detected using the hypoxia marker pimonidazole hydrochloride (Hypoxyprobe‐1, Natural Pharmacia Inc., Belmont, MA, USA). Pimonidazole (60 mg kg^−1^) was injected intravenously 1 h before harvesting the tissues of the plaque‐bearing rabbits. Immunofluorescence was subsequently performed, and the hypoxic area in the plaque region was measured using the IPP software.

### Histopathological Analysis and Atherosclerotic Lesion Quantification

Forty‐two plaque‐bearing rabbits were randomly assigned to the control, LIFU, PFP–HMME@PLGA/MnFe_2_O_4_–Ram, PFP@PLGA/MnFe_2_O_4_–Ram + LIFU, PFP–HMME@PLGA/MnFe_2_O_4_ + LIFU, and PFP–HMME@PLGA/MnFe_2_O_4_–Ram + LIFU groups (*n* = 7 per group). On day 28 after treatment, the rabbits were sacrificed, and the right femoral arteries containing advanced plaques were harvested and cut into three segments. H&E, Prussian blue, Oil Red, and Masson's trichrome staining were performed. For immunohistochemical staining, the sections were stained with the following primary antibodies: anti‐CD31 (1:20; Abcam, Cat#ab9498), anti‐RAM‐11 (1:1200, Dako, Cat#M0633), anti‐HIF‐1*α* (1:200, Bioss, Shanghai, China; Cat#bs‐0737R), and anti‐*α*‐actin (1:2000, Sigma, Cat#A2547), followed by HRP‐conjugated secondary antibodies (1:250; Life Technologies, Carlsbad, CA, USA). All antigens were detected using standard peroxidase antiperoxidase techniques and diaminobenzidine substrate chromogens. All histopathological parameters and endothelialization scores were quantitatively measured, as previously described.^[^ ^16^, ^35^ ^]^

### ^18^FDG‐PET/CT Imaging

Twenty‐four plaque‐bearing rabbits were randomly assigned to the control, LIFU, PFP–HMME@PLGA/MnFe_2_O_4_–Ram, PFP@PLGA/MnFe_2_O_4_–Ram + LIFU, PFP–HMME@PLGA/MnFe_2_O_4_ + LIFU, and PFP–HMME@PLGA/MnFe_2_O_4_–Ram + LIFU groups (*n* = 4 each). On day 28 after treatment, the rabbits were fasted overnight. Subsequently, ^18^FDG (0.1 mCi kg^−1^) was injected intravenously 1 h before the right femoral arteries containing advanced plaques were harvested and cut into two segments. ^18^FDG‐PET/CT imaging of the femoral arteries was performed using a nanoScan PET/CT scanner (Mediso, USA). For PET/CT imaging in 3D mode, the arteries were covered for 20 min in a single bed position, and the total standardized uptake value (SUV) was measured using the PMOD software.

### Pharmacokinetic Study

Plaque‐bearing rabbits (*n* = 3 for each group) were administered a single dose of PFP–HMME@PLGA/MnFe_2_O_4_–Ram or PFP–HMME@PLGA/MnFe_2_O_4_ NPs (1 mg mL^−1^, 50 mL for each). The rabbits were subjected to overnight fasting, and the blood (1 mL at each time point) was collected through a capillary glass tube from the postglomus venous plexus before injection and at 0.03, 0.08, 0.17, 0.5, 1, 3, 5, 8, 12, and 24 h after injection. For pharmacokinetic analysis, ICP‐OES was used to measure the Fe and Mn concentrations in the plasma. The concentration–time curve was determined using Origin‐Pro 7.0, software (OriginLab, Northampton, MA, USA).

### In Vivo Biocompatibility Studies

Five healthy rabbits were intravenously injected with PFP–HMME@PLGA/MnFe_2_O_4_–Ram NPs (1 mg mL^−1^, 50 mL each). Blood samples were collected for routine blood tests and biochemical and coagulation examinations before injection, and on day 1, day 7, and day 28 after injection. The healthy rabbits were sacrificed at each time point (*n* = 3), and the major organs (heart, liver, spleen, lung, and kidney) were harvested for H&E staining to evaluate the biosafety of NPs. The plaque‐bearing rabbits were randomly assigned to the control, LIFU, PFP–HMME@PLGA/MnFe_2_O_4_–Ram, PFP@PLGA/MnFe_2_O_4_–Ram + LIFU, PFP–HMME@PLGA/MnFe_2_O_4_ + LIFU, and PFP–HMME@PLGA/MnFe_2_O_4_–Ram + LIFU groups. Before treatment and on day 28 after treatment, blood samples were collected for routine blood and biochemical examinations (*n* = 4). Before treatment and on day 3, day 7, day 14, and day 28 after treatment, body weight was measured for the indicated groups (*n* = 5). On day 28 after treatment, the plaque‐bearing rabbits were sacrificed, and the major organs (heart, liver, spleen, lung, and kidney) were harvested for H&E staining to evaluate the biosafety of the intervention.

### Endothelial Cell Function Assessment

Eight rabbits with advanced femoral plaques were randomly assigned to the control and PFP–HMME@PLGA/MnFe_2_O_4_–Ram + LIFU groups (*n* = 4). The ultrasound scanning used to assess FMD was performed as described previously^[^ ^36^ ^]^ at baseline and 28 days after PFP–HMME@PLGA/MnFe_2_O_4_–Ram‐mediated SDT treatment. After anesthesia, the rabbits were fixed in the supine position and connected to an electrocardiogram (ECG). The lumen diameter of the right femoral artery was examined longitudinally and measured using 2D ultrasound images, with a 21 MHz linear array transducer (Vevo LAZR, Canada). Depth and gain settings were set to optimize images of the lumen/arterial wall interface, and the machine operating parameters were not changed during any study. The diameter was measured by two experienced observers who were unaware of the experimental details, at a fixed distance from an anatomical marker, such as a bifurcation, with ultrasound calipers. The diameter at rest (*D*_0_) was then recorded. The increased flow was then induced by inflation of a pneumatic tourniquet to a pressure of 280 mmHg for 5 min. A second scan was taken 30–90 s after cuff deflation, and the diameter was recorded as *D*_1_. The FMD was calculated as follows: FMD% = (*D*_1_ − *D*_0_)/*D*_0_ × 100%. At day 28 after treatment, the rabbits were sacrificed, and the cryosections were stained with anti‐CD31 and VCAM‐1 (1:500, Cat#bs‐0920R, Bioss, China) antibodies. After overnight incubation at 4 °C, the sections were washed three times with PBS and incubated with FITC‐conjugated antirabbit IgG and TRITC‐conjugated antimouse IgG for 1 h at room temperature. The nuclei were counterstained with DAPI, and the sections were visualized under a fluorescence microscope. VCAM‐1‐positive areas that colocalized with DAPI and CD31 were counted as positive and measured using IPP software.

### Statistical Analysis

All quantitative data were expressed as the mean ± standard deviation (SD). Statistical analysis was performed using GraphPad 6.0 (La Jolla, CA, USA). Differences between the two groups were analyzed using Student's unpaired or paired *t*‐tests. Differences among multiple groups were calculated by one‐way analysis of variance (ANOVA), and one‐way or two‐way ANOVA with repeated measures with Tukey's or Dunnett's post‐hoc test. The statistical tests were two‐sided, and a value of *p* < 0.05 was considered statistically significant. All experiments were performed using *n* ≥ 3 biological replicates.

## Conflict of Interest

The authors declare no conflict of interest.

## Author Contributions

J.Y., Y.S., and H.R. contributed in project conceptualization and supervision; J.Y., Z.Y., L.H., C.Y., J.W., L.Z., J.Z., and Y.S. contributed in investigation; J.Y., Y.C., L.H., P.L., and Z.W. contributed in data curation; J.Y., and Y.S. contributed in methodology and formal analysis and visualization; J.Y. contributed in writing the original draft; Y.S. and H.R. contributed in writing, reviewing, and editing; Y.S. and H.R. contributed in funding acquisition.

## Supporting information

Supporting Information

Supplemental Table 1

## Data Availability

The data that support the findings of this study are available from the corresponding author upon reasonable request.
