# Supplementary material for: Low‐Intensity Focused Ultrasound‐Responsive Ferrite‐Encapsulated Nanoparticles for Atherosclerotic Plaque Neovascularization Theranostics
Source: Adv Sci (Weinh). 2021 Aug 11;8(19):2100850. doi: 10.1002/advs.202100850 (PMC8498883; doi:10.1002/advs.202100850)
Supplement: Supplementary file 1 — Supporting Information [file ADVS-8-2100850-s002.pdf]

© 2021 Wiley-VCH GmbH

ADVANCED SCIENCE
Open Access

Supporting Information

for *Adv. Sci.*, DOI: 10.1002/advs.202100850

Low-Intensity Focused Ultrasound-Responsive Ferrite-encapsulated Nanoparticles for Atherosclerotic Plaque Neovascularization Theranostics

Jianting Yao, Zhuowen Yang, Liandi Huang, Chao Yang, Jianxin Wang, Yang Cao, Lan Hao, Liang Zhang, Jingqi Zhang, Pan Li, Zhigang Wang, Yang Sun, and Haitao Ran**

Supporting Information

Low-Intensity Focused Ultrasound-Responsive Ferrite-encapsulated Nanoparticles for Atherosclerotic Plaque Neovascularization Theranostics

Jianting Yao, Zhuowen Yang, Liandi Huang, Chao Yang, Jianxin Wang, Yang Cao, Lan Hao, Liang Zhang, Jingqi Zhang, Pan Li, Zhigang Wang, Yang Sun, Haitao Ran**

Dr. J Yao, Prof. Y Cao, Prof. L Hao, Dr. L Zhang, Prof. P Li, Prof. Z Wang, Prof. Y Sun, Prof. H Ran
Department of Ultrasound, Chongqing Key Laboratory of Ultrasound Molecular Imaging, the Second Affiliated Hospital of Chongqing Medical University, Chongqing 400010, P. R. China

Dr. Z Yang, Dr. J Zhang
Department of Cardiology, the First Affiliated Hospital, Cardiovascular Institute, Harbin Medical University, Harbin 150001, P. R. China

Dr. L Huang
State Key Laboratory of Ultrasound in Medicine and Engineering, the Second Affiliated Hospital of Chongqing Medical University, Chongqing 400010, P. R. China

Dr. C Yang
Department of Radiology, Chongqing General Hospital, University of Chinese Academy of Sciences, Chongqing 400014, P. R. China

Dr. J Wang
Department of Ultrasound, the First Affiliated Hospital of Harbin Medical University, Harbin 150001, P. R. China

Prof. Yang Sun
Department of Ultrasound, Chongqing Key Laboratory of Ultrasound Molecular Imaging.The Second Affiliated Hospital of Chongqing Medical University, Chongqing 400010, P. R. China. Tel.: +86-023-68485555; Email: sunyang@hospital.cqmu.edu.cn

Prof. Haitao Ran
Department of Ultrasound, Chongqing Key Laboratory of Ultrasound Molecular Imaging.The Second Affiliated Hospital of Chongqing Medical University, Chongqing 400010, P. R. China. Tel.: +86-023-68485555; Email: ranhaitao@cqmu.edu.cn

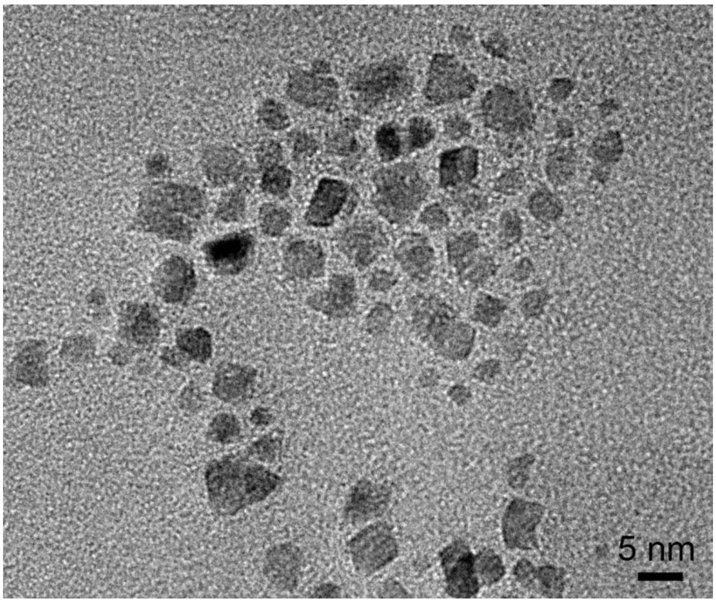

Figure S1. Transmission electron microscopy image of 3 nm $MnFe_2O_4$.

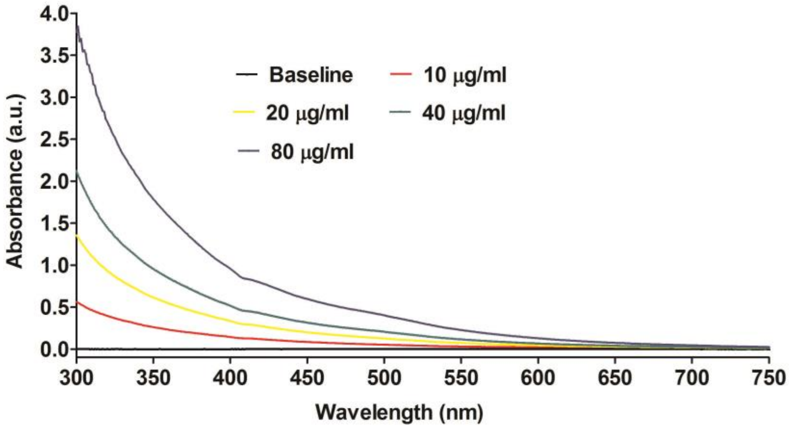

Figure S2. UV–vis absorbance spectra of 3 nm $MnFe_2O_4$ at elevated concentrations.

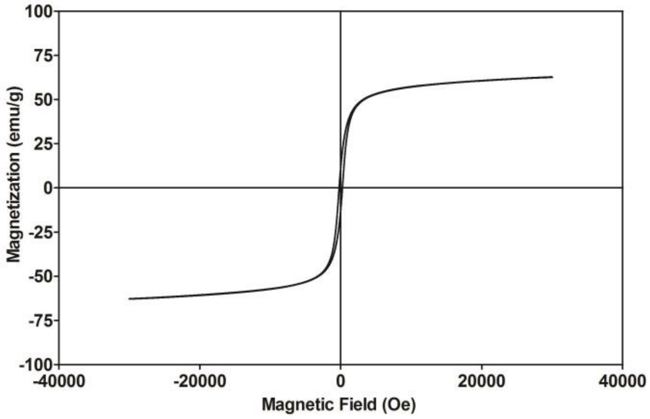

Figure S3. Magnetization hysteresis loops of 3 nm $MnFe_2O_4$ at 300 K ranging from −30 kOe to +30 kOe.

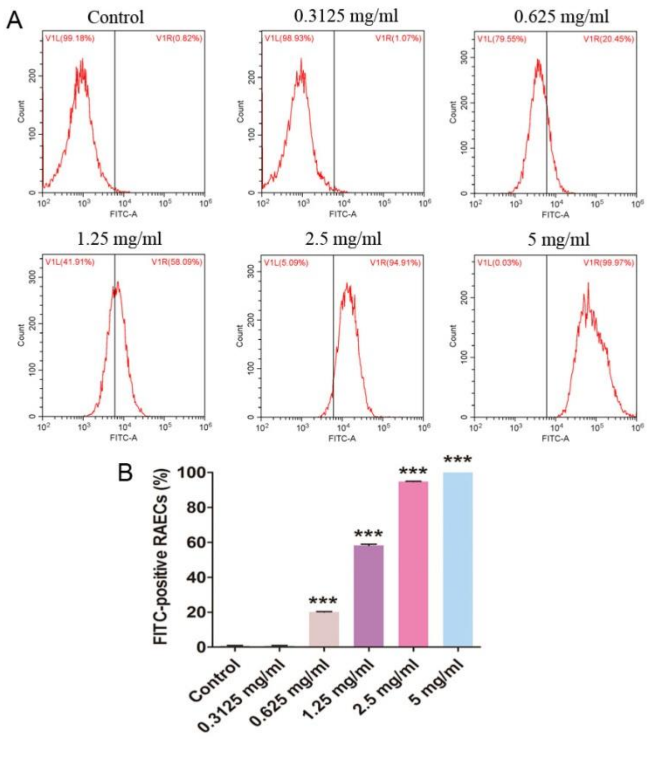

Figure S4. Cell−targeting behavior of ramucirumab. After incubating rabbit aortic endothelial cells (RAECs) with FITC-labeled ramucirumab for 3 h, the cells were harvested and analyzed using flow cytometry. **(A)** Representative cytograms and **(B)** quantification of the FITC-positive RAECs (n = 3). Data are shown as the means ± SD. (B) ANOVA with Dunnett's post-hoc test. *** $p < 0.001$ vs. control.

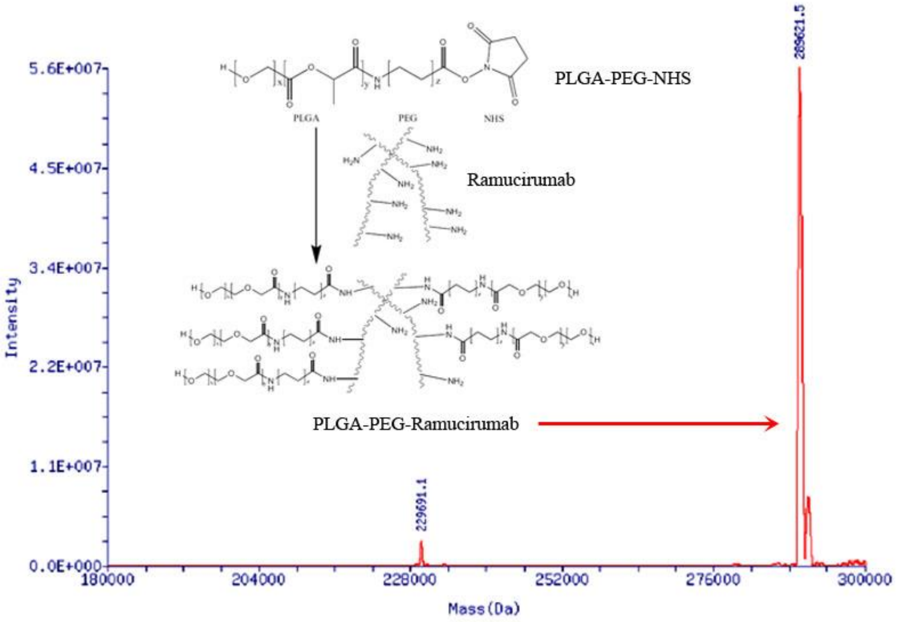

Figure S5. Mass spectrum of PLGA-PEG-ramucirumab.
PLGA-PEG-ramucirumab was synthesized using the carbodiimide method from PLGA-PEG-NHS and ramucirumab and identified by mass spectroscopy.

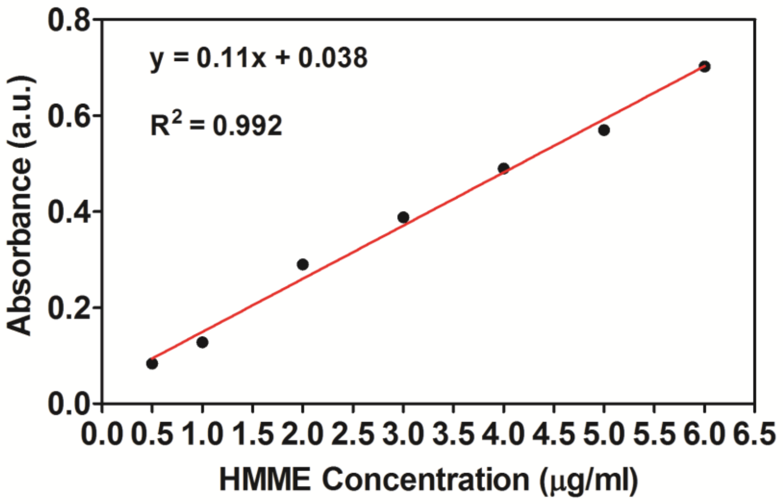

Figure S6. Standard curve of HMME based on UV–Vis–NIR absorbance spectra.

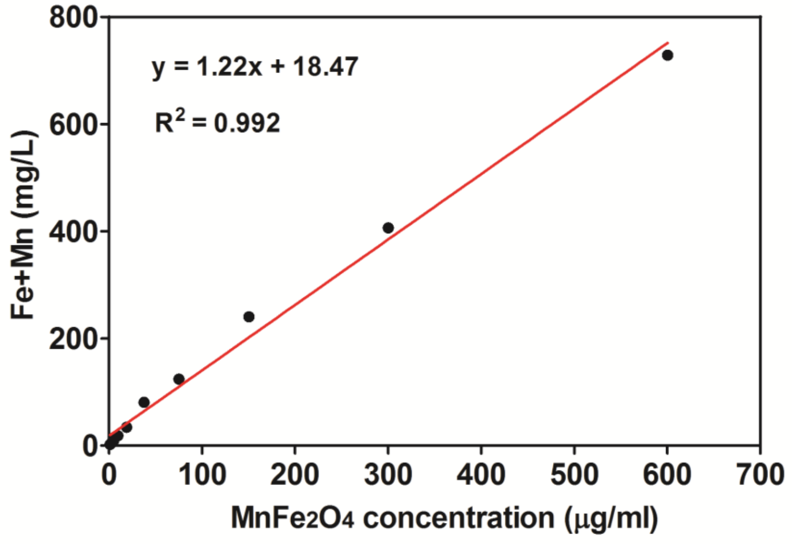

Figure S7. Standard curve of 3 nm $MnFe_2O_4$ based on inductively coupled plasma optical emission spectrometry (ICP-OES).

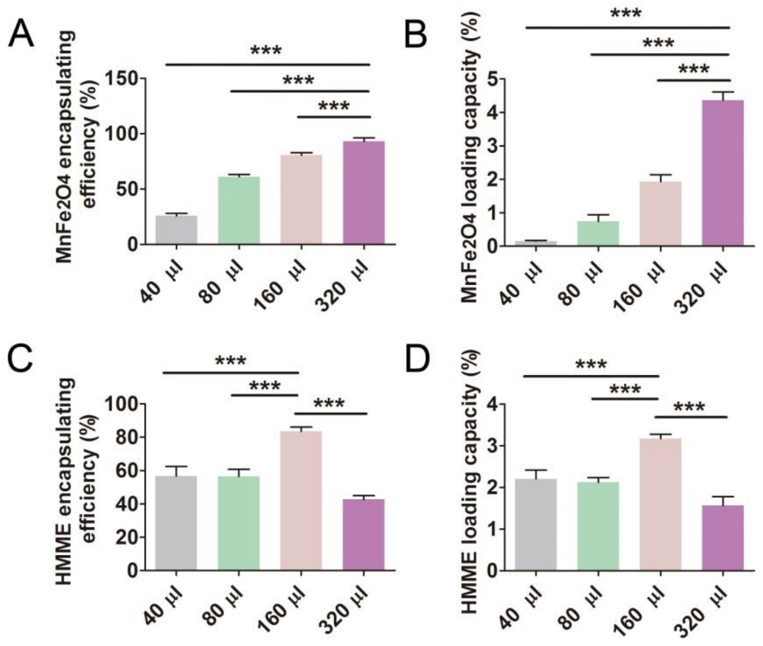

Figure S8. Encapsulating efficiency and loading capacity of $MnFe_2O_4$ **(A - B)** and HMME **(C - D)** in the PFP-HMME@PLGA/$MnFe_2O_4$-Ram NPs with different initial $MnFe_2O_4$ loadings (40, 80, 160, and 320 μL) (n = 3). Data are shown as the means ± SD. (A - D) ANOVA with Dunnett's post-hoc test. *** $p < 0.001$.

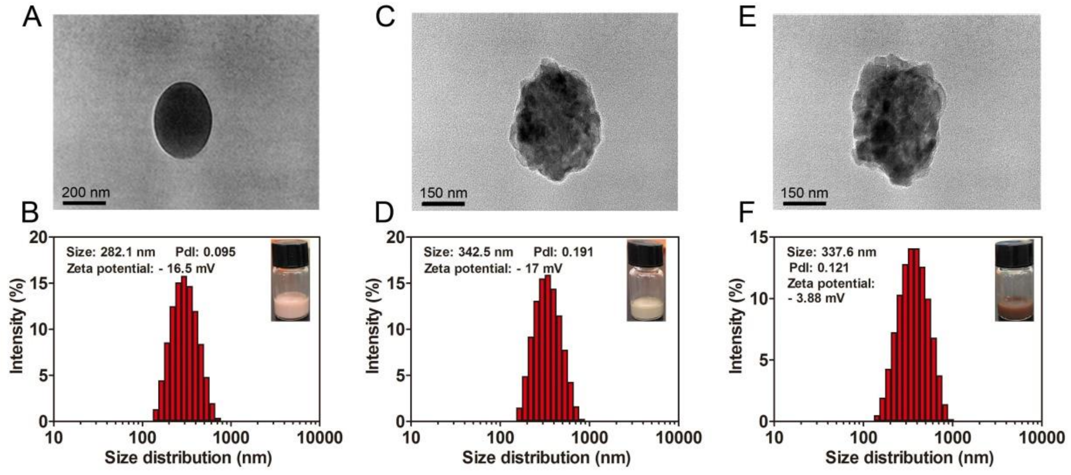

Figure S9. The TEM, size distribution, zeta potential, and digital images of **(A - B)** PFP-HMME@ PLGA-Ram, **(C - D)** PFP@PLGA/$MnFe_2O_4$-Ram, and **(E - F)** PFP-HMME@PLGA/$MnFe_2O_4$ NPs.

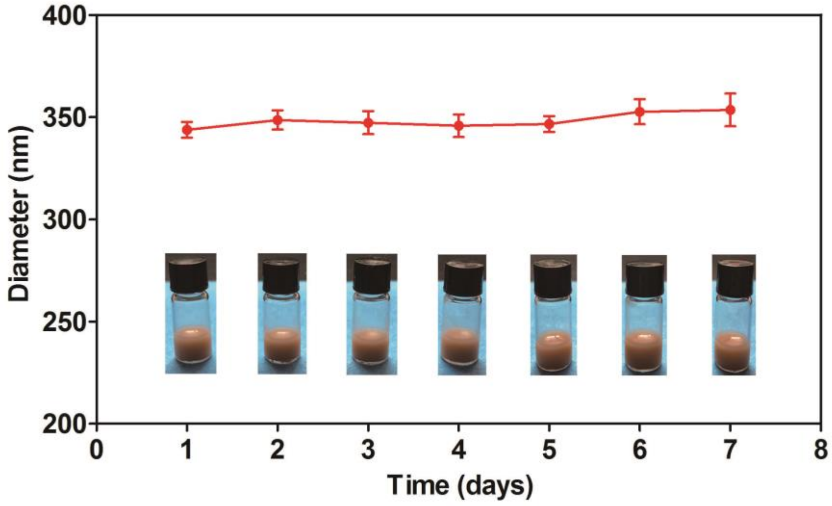

Figure S10. The size distribution of the PFP-HMME@PLGA/$MnFe_2O_4$-Ram NPs within 7 days (n = 3)**.** Inset: the corresponding digital photos of the NPs dispersed in PBS (10 mg mL^{-1}). Data are shown as the means ± SD. ANOVA with Dunnett's post-hoc test.

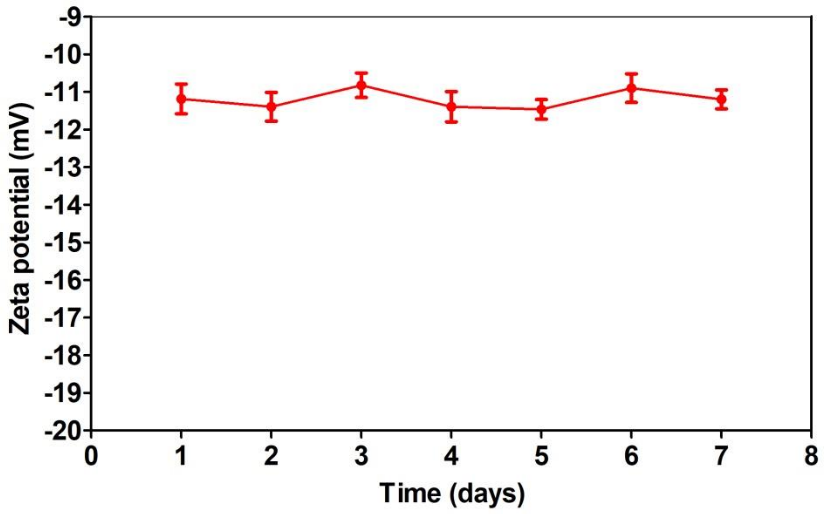

Figure S11. The zeta potential of the PFP-HMME@PLGA/$MnFe_2O_4$-Ram NPs dispersed in PBS (10 mg mL^{-1}) within 7 days (n = 3)**.** Data are shown as the means ± SD. ANOVA with Dunnett's post-hoc test.

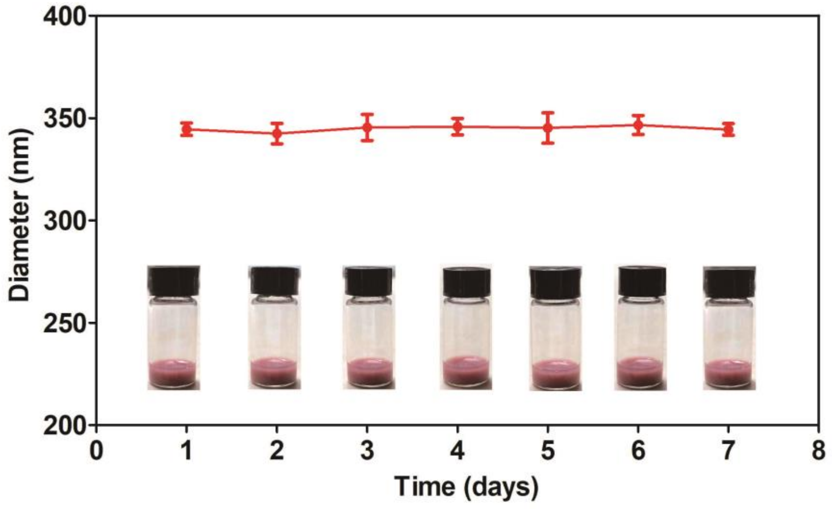

Figure S12. The size distribution of the PFP-HMME@PLGA/$MnFe_2O_4$-Ram NPs within 7 days (n = 3)**.** Inset: the corresponding digital photos of the NPs dispersed in 5% (vol/vol) FBS RPMI-1640 culture medium (10 mg mL^{-1}). Data are shown as the means ± SD. ANOVA with Dunnett's post-hoc test.

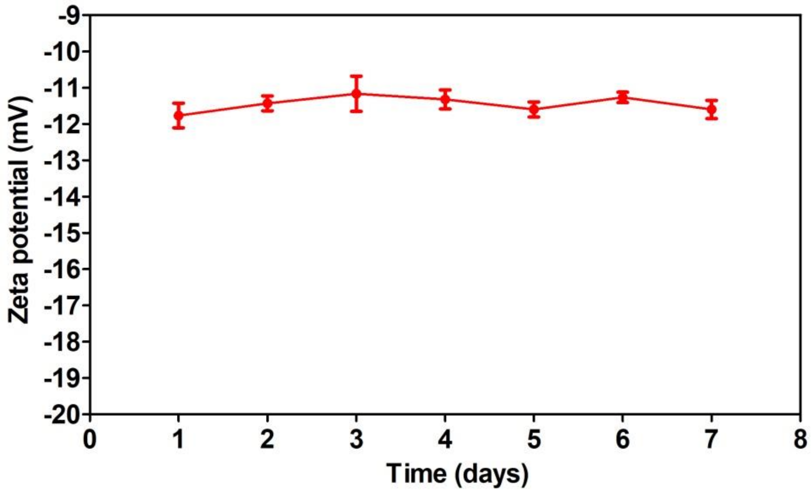

Figure S13. The zeta potential of the PFP-HMME@PLGA/$MnFe_2O_4$-Ram NPs dispersed in 5% (vol/vol) FBS RPMI-1640 culture medium (10 mg mL^{-1}) within 7 days (n = 3)**.** Data are shown as the means ± SD. ANOVA with Dunnett's post-hoc test.

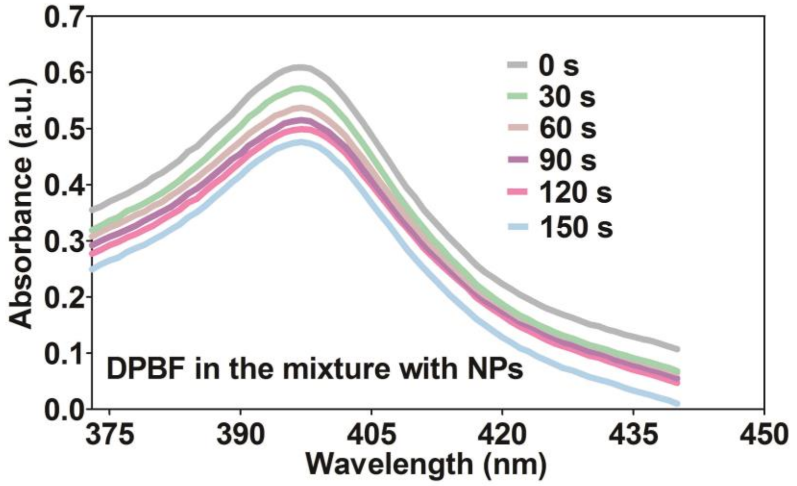

Figure S14. A mixture of DPBF (50 µM) and PFP-HMME@PLGA/$MnFe_2O_4$-Ram NPs (125 µg mL^{-1}) was irradiated by LIFU (1.5 W cm^{-2}, 1 MHz) for 0, 30, 60, 90, 120, and 150 s. Subsequently, the absorbance spectra of DPBF were determined by UV-vis spectrophotometer.

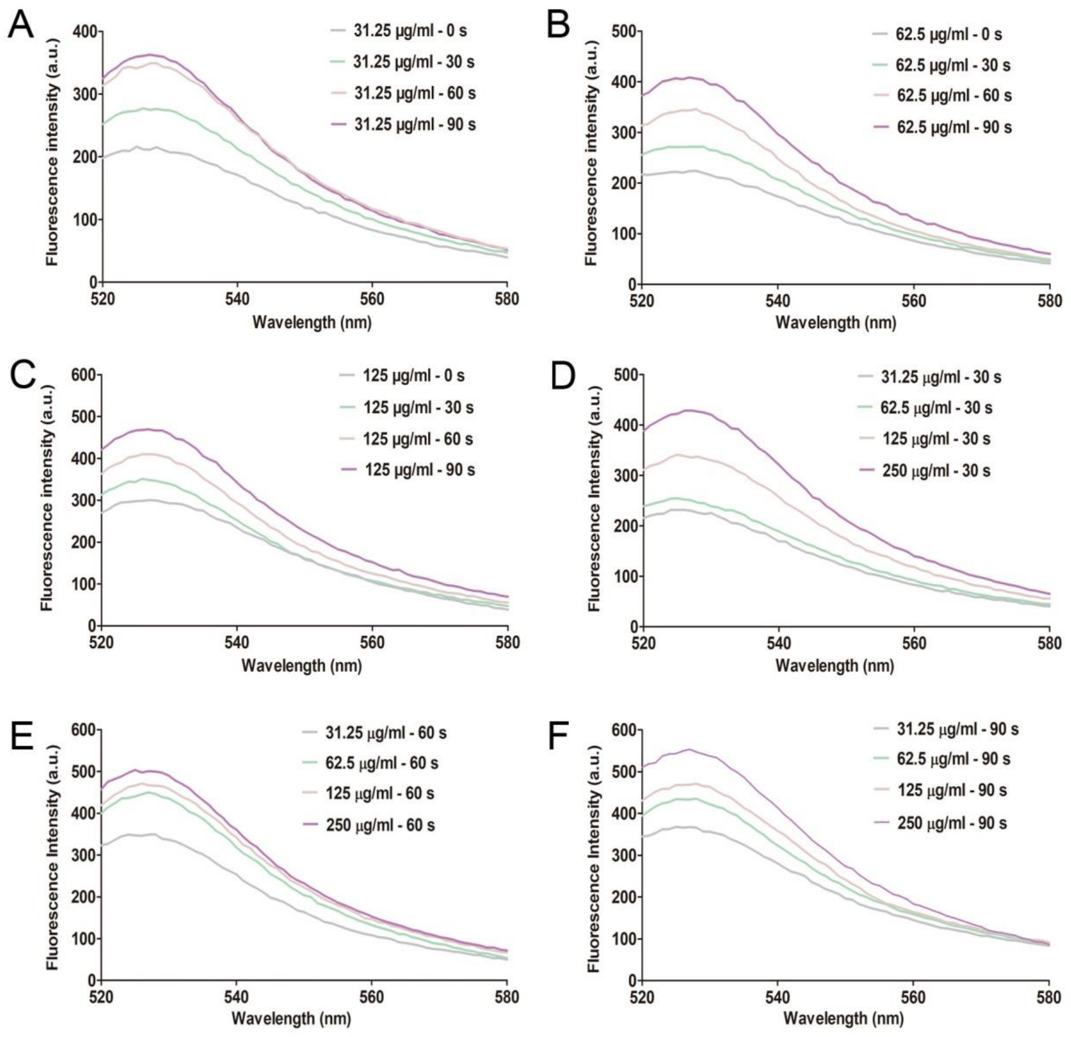

Figure S15. SOSG fluorescence intensity of PFP-HMME@PLGA/$MnFe_2O_4$-Ram NPs. (A - C) Concentration-dependent and **(D - F)** time-dependent ROS production of the NPs, following irradiation with LIFU (1.5 W cm^{-2}, 1 MHz).

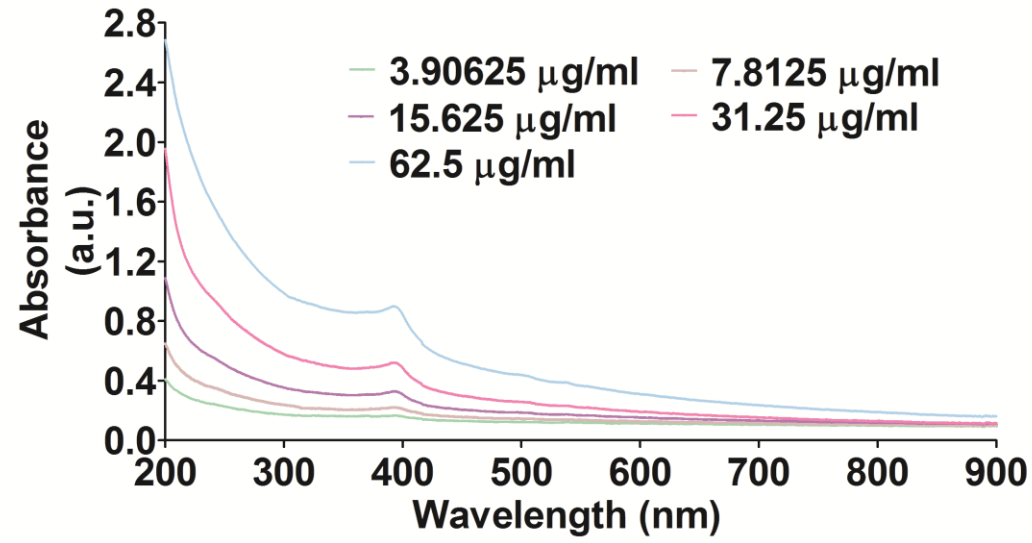

Figure S16. UV–Vis–NIR absorbance spectra of the PFP-HMME@PLGA/ $MnFe_2O_4$-Ram NPs at elevated concentrations.

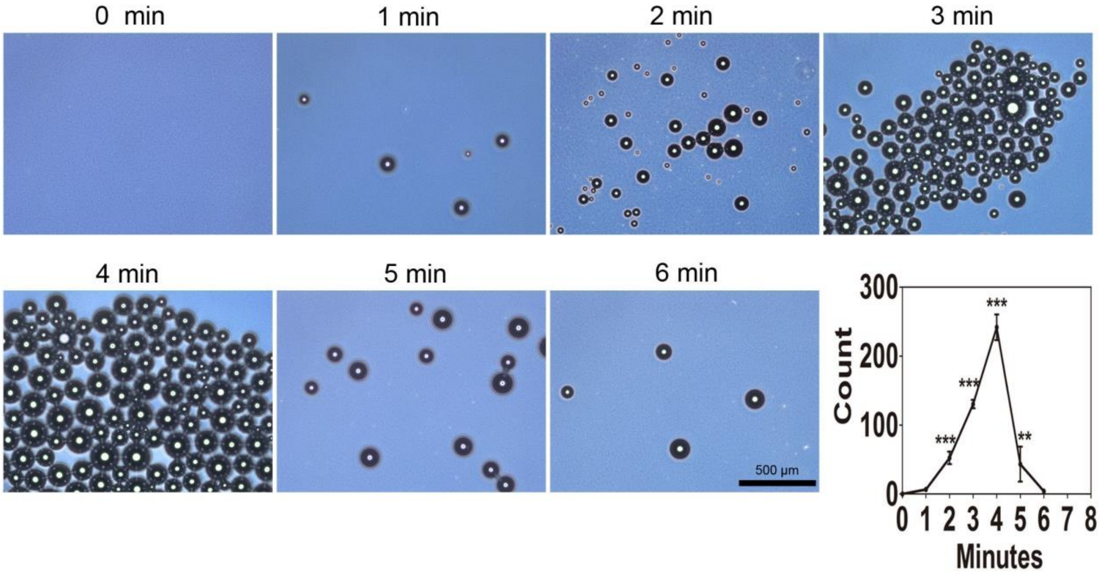

Figure S17. In vitro LIFU-responsive phase transition of PFP-HMME@PLGA/ $MnFe_2O_4$-Ram NPs. Microscopic images of the PFP phase transition after LIFU (1.5 W cm^{-2}, 1 MHz) irradiation at different time points and the corresponding number of the PFP microbubbles (n = 3). Data are shown as the means ± SD. ANOVA with Dunnett's post-hoc test. ** $p < 0.01$, *** $p < 0.001$ vs. 0 min.

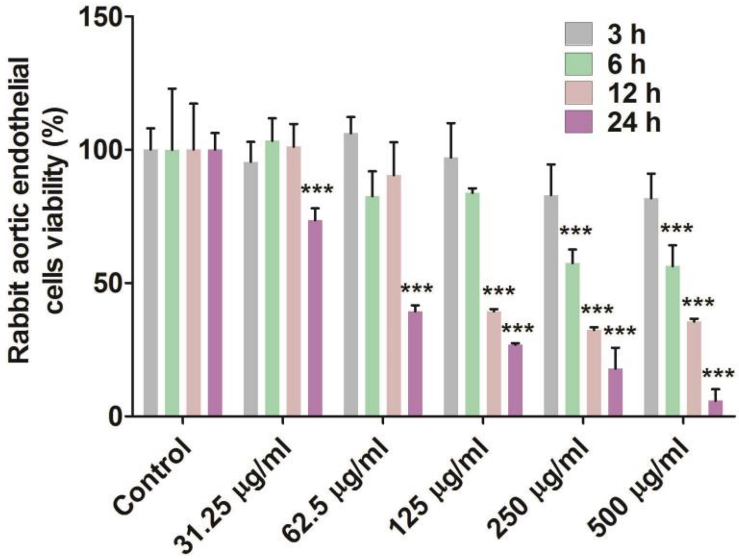

Figure S18. Cell viability of rabbit aortic endothelial cells (RAECs) treated with different concentrations (31.25, 62.5, 125, 250, and 500 μg mL^{-1}) of the PFP-HMME @PLGA/$MnFe_2O_4$-Ram NPs for 3, 6, 12 and 24 h detected using the CCK-8 assay (n = 5). Data are shown as the means ± SD. ANOVA with Dunnett's post-hoc test. *** p < 0.001 vs. control.

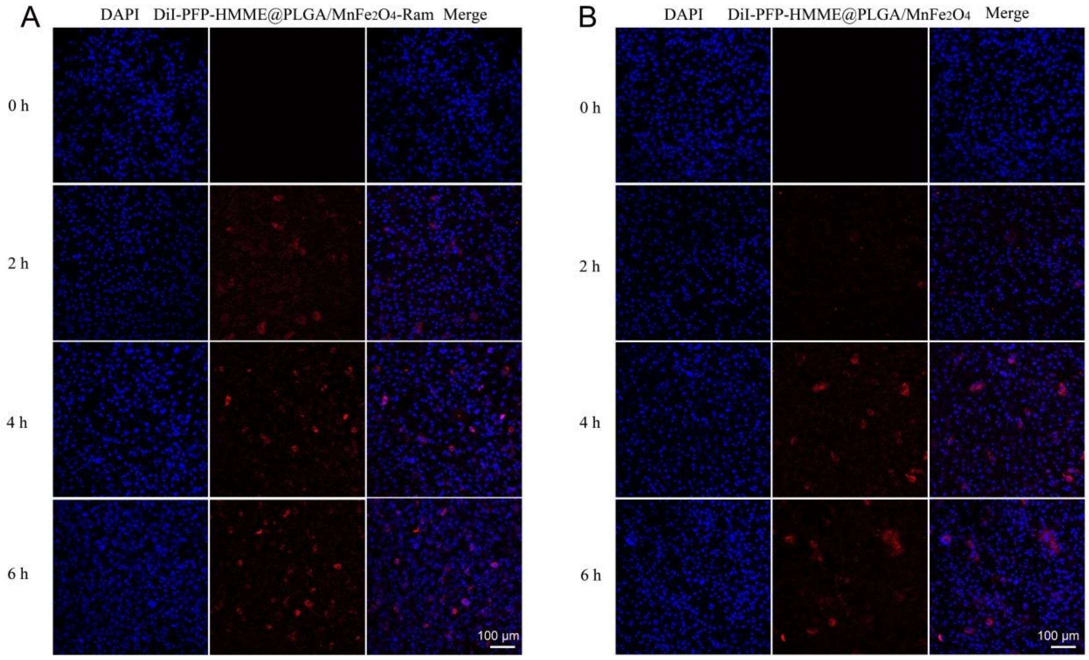

Figure S19. Cellular uptake of DiI-labeled **(A)** PFP-HMME@PLGA/$MnFe_2O_4$-Ram NPs and **(B)** PFP-HMME@PLGA/$MnFe_2O_4$ NPs in rabbit aortic endothelial cells (RAECs) as observed using confocal microscopy after 0, 2, 4, and 6 h incubation.

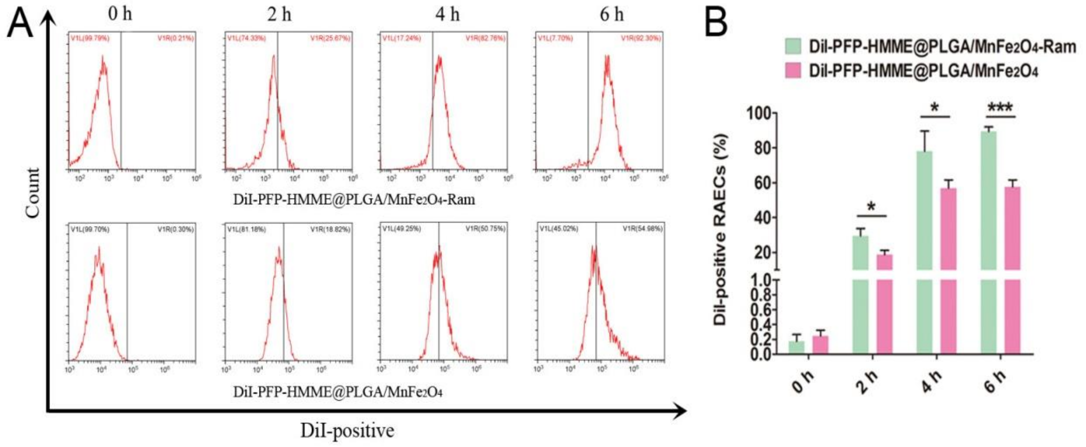

Figure S20. Flow cytometry analysis of DiI-labeled PFP-HMME@PLGA/ $MnFe_2O_4$-Ram NPs and PFP-HMME@PLGA/$MnFe_2O_4$ NPs in rabbit aortic endothelial cells (RAECs). (A) Representative cytograms and **(B)** quantification of the DiI-positive RAECs (n = 3). Data are shown as the means ± SD. (B) Student's unpaired *t*-test. * $p < 0.05$, *** $p < 0.001$.

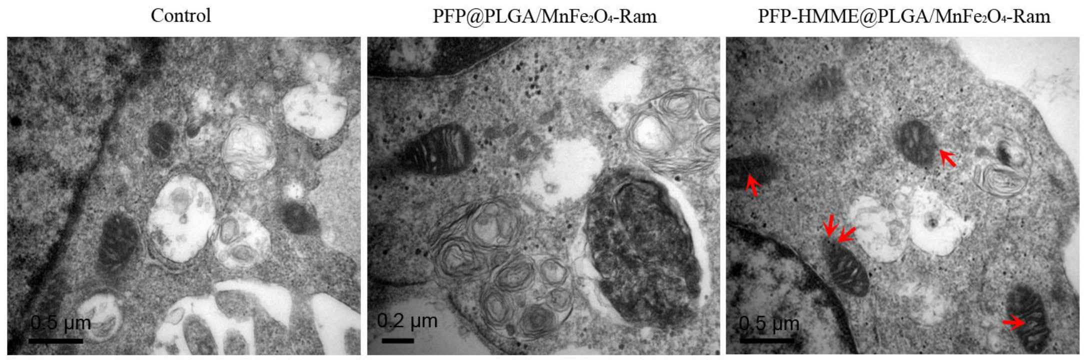

Figure S21. TEM images of rabbit aortic endothelial cells (RAECs) incubated with the PFP@PLGA/$MnFe_2O_4$-Ram NPs and PFP-HMME@PLGA/$MnFe_2O_4$ -Ram nanoparticles (NPs). After RAECs were incubated with PFP@PLGA/ $MnFe_2O_4$-Ram NPs and PFP-HMME@PLGA/ $MnFe_2O_4$-Ram NPs for 2 h, the NPs colocalized with mitochondria were observed. Red arrows indicate the PFP-HMME @PLGA/$MnFe_2O_4$-Ram NPs accumulated in the mitochondria of the RAECs.

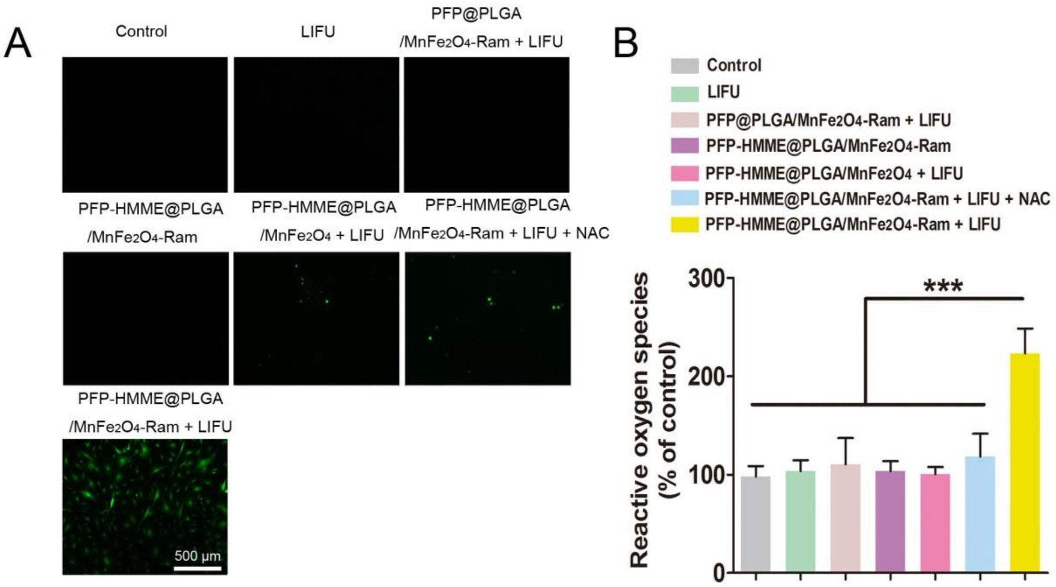

Figure S22. PFP-HMME@PLGA/$MnFe_2O_4$-Ram NP-mediated SDT induces ROS production in rabbit aortic endothelial cells (RAECs). After RAECs were incubated with NPs (31.25 μg mL^{-1}) for 2 h, ROS generation using DCFH-DA in the indicated groups were observed using confocal microscopy **(A)** and quantified on a microplate reader **(B)** (n = 10). (B) Data are shown as the means ± SD. ANOVA with Dunnett's post-hoc test. *** p < 0.001.

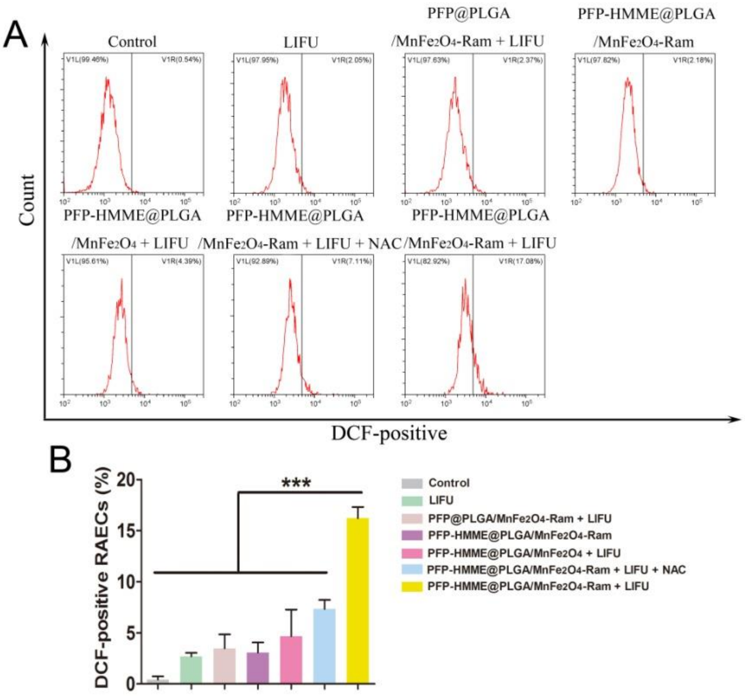

Figure S23. PFP-HMME@PLGA/$MnFe_2O_4$-Ram NP-mediated SDT induces ROS production in rabbit aortic endothelial cells (RAECs). After RAECs were incubated with the NPs (31.25 μg mL^{-1}) for 2 h, the cells were harvested and ROS generation using DCFH-DA was analyzed using flow cytometry. Representative cytograms **(A)** and quantification of the DCF-positive RAECs **(B)** (n = 3). Data are shown as the means ± SD. (B) ANOVA with Dunnett's post-hoc test. *** $p < 0.001$.

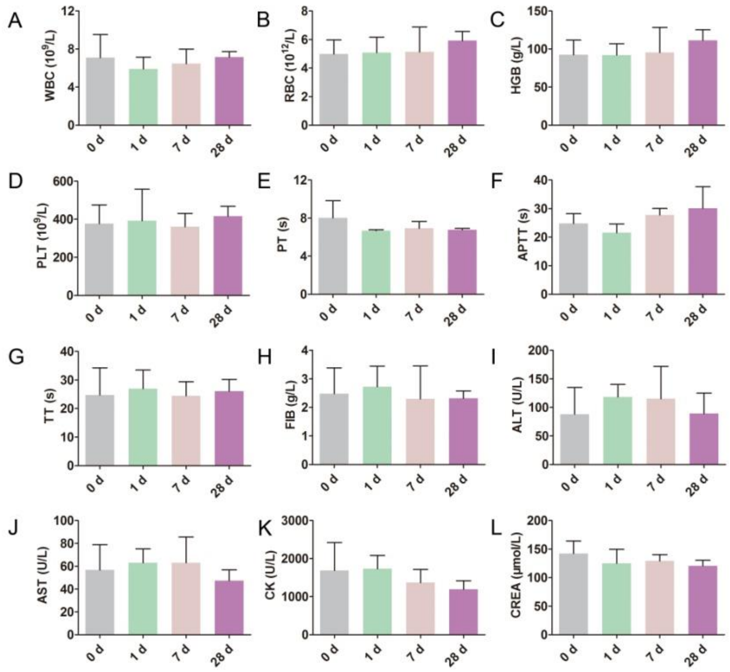

Figure S24. Blood examination of five healthy rabbits after 0, 1, 7, and 28 days of intravenous exposure to PFP-HMME@PLGA/$MnFe_2O_4$-Ram NPs (n = 5). Data are shown as the means ± SD. (A - L) one-way ANOVA with repeated measures with Tukey's post-hoc test.

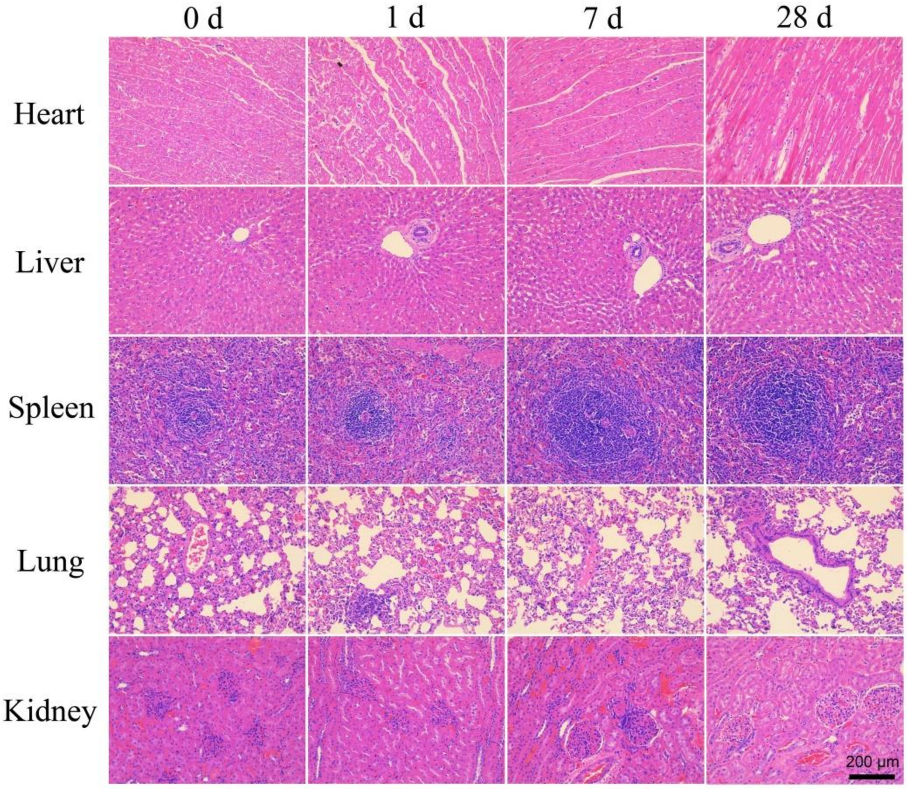

Figure S25. Histopathological images of the organs of healthy rabbits after 0, 1, 7, and 28 days of intravenous exposure to PFP-HMME@PLGA/$MnFe_2O_4$-Ram NPs.

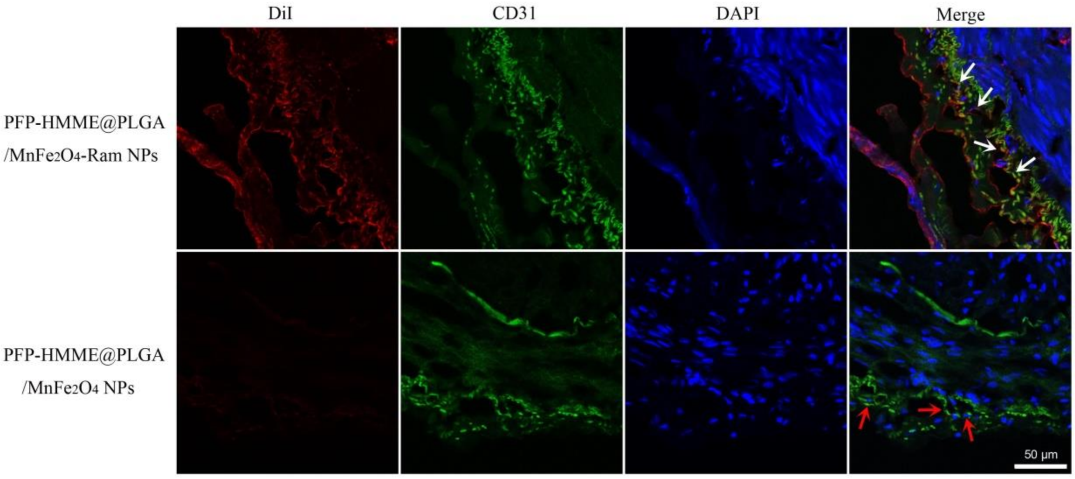

Figure S26. In vivo neovessel-targeting behavior of PFP-HMME@PLGA/ $MnFe_2O_4$-Ram NPs in the rabbit advanced plaque. The immunofluorescence images of plaque-bearing rabbits after intravenous injection of DiI-labeled targeted NPs and non-targeted NPs at 90 min. White arrows indicate CD31-positive neovessels colocalized with DiI-labeled PFP-HMME@PLGA/$MnFe_2O_4$-Ram NPs; red arrows indicate CD31-positive neovessels.

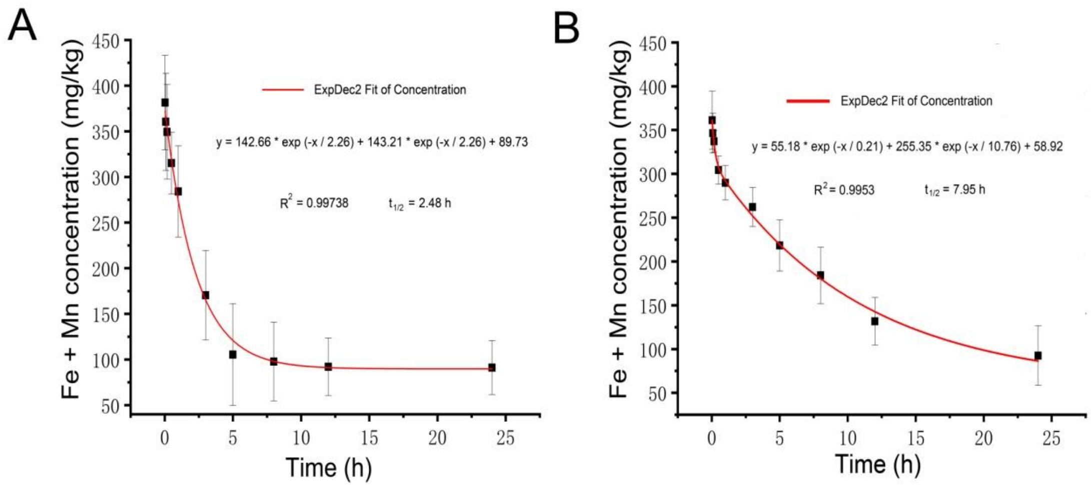

Figure S27. Plasma Fe + Mn concentration–time curve after a single administration of PFP-HMME@PLGA/$MnFe_2O_4$ NPs **(A)** and PFP-HMME@PLGA/$MnFe_2O_4$-Ram NPs **(B)** to rabbits (n = 3).

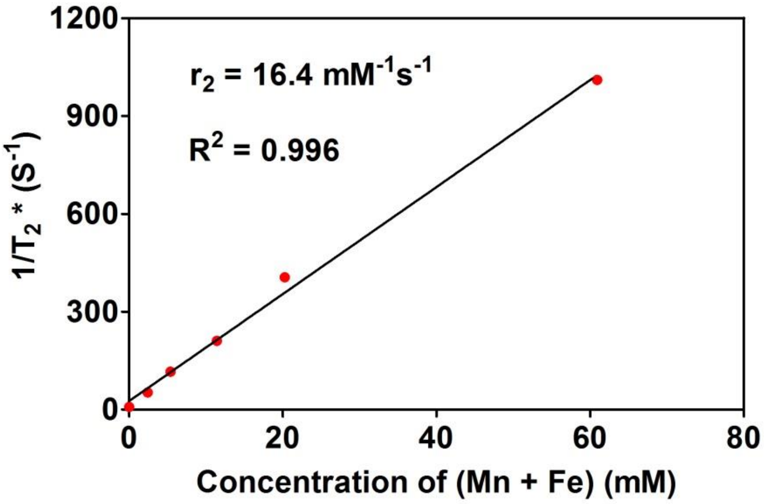

Figure S28. In vitro MRI T2 contrast images and R_2* value of PFP-HMME @PLGA/$MnFe_2O_4$-Ram dissolved in PBS at different Mn + Fe concentrations (0.014, 2.432, 5.400, 11.415, 20.260, and 60.917 mM).

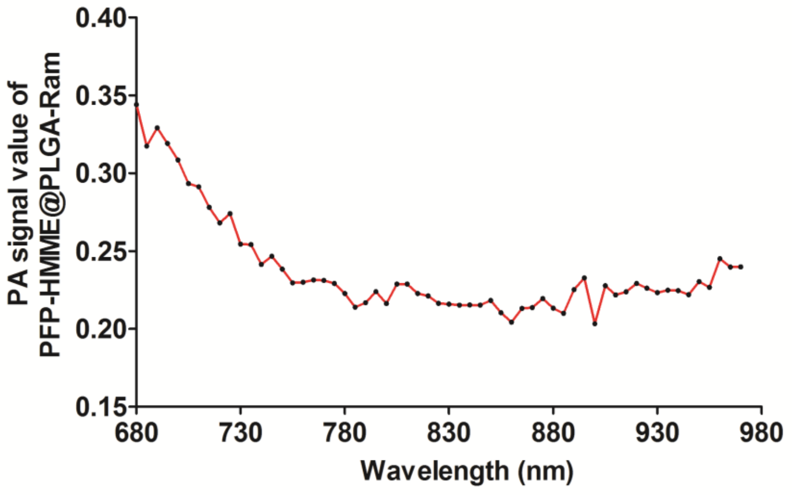

Figure S29. Photoacoustic intensity (PA) of the PFP-HMME@PLGA-Ram NPs under full-spectrum scanning (ranging from 680 to 970 nm).

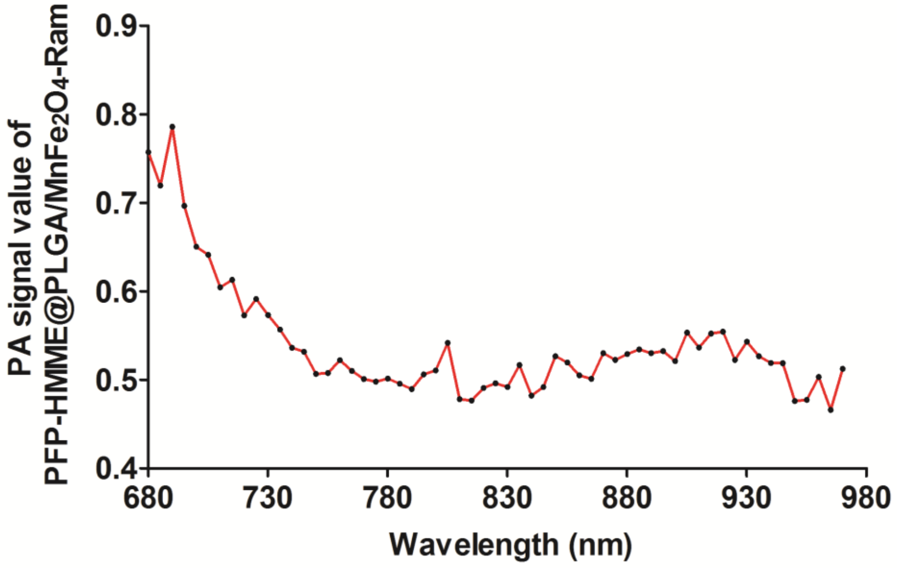

Figure S30. Photoacoustic intensity (PA) of the PFP-HMME@PLGA/ $MnFe_2O_4$-Ram NPs under full-spectrum scanning (ranging from 680 to 970 nm).

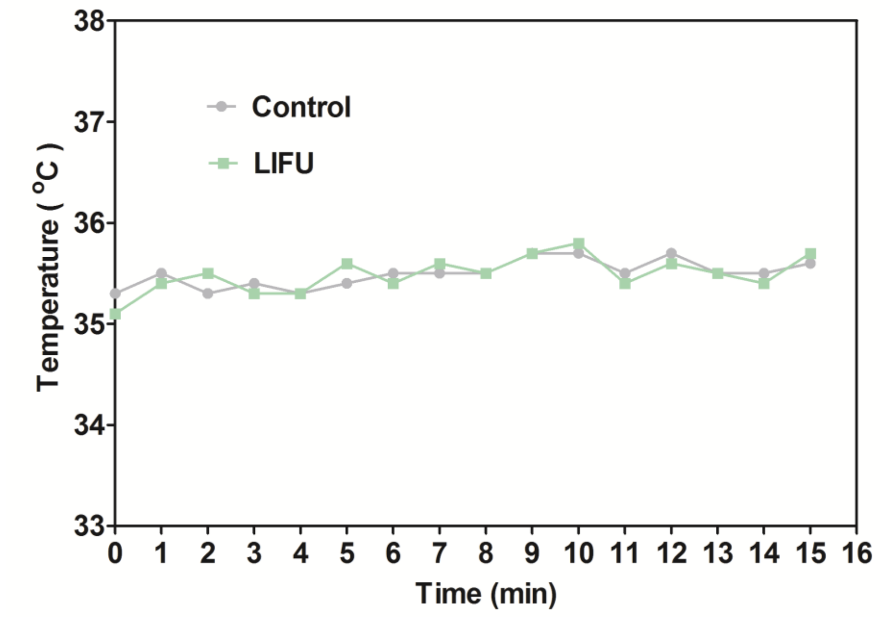

Figure S31. Thermal effects of LIFU irradiation on the right femoral artery region of the normal rabbit. An infrared thermal imaging camera was used to monitor the local temperature changes during LIFU irradiation (1.5 W cm^{-2}, 1 MHz, 15 min). Data on graph are representative of three independent experiments.

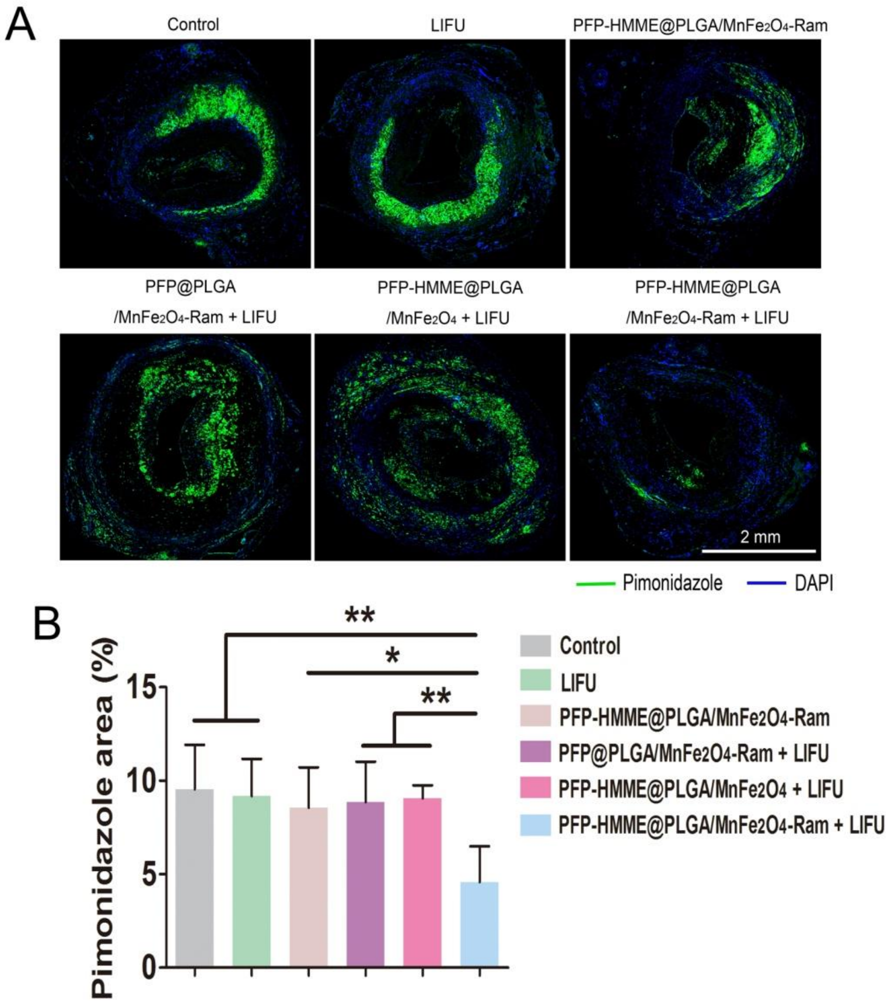

Figure S32. PFP-HMME@PLGA/$MnFe_2O_4$-Ram NP-mediated SDT decreases hypoxia in the rabbit femoral advanced plaque on day 3 after treatment. (A) Representative sections and **(B)** quantification of the hypoxic area in plaque from different groups (n = 5). Data are shown as the means ± SD. (B) ANOVA with Dunnett's post-hoc test. * $p < 0.05$, ** $p < 0.01$.

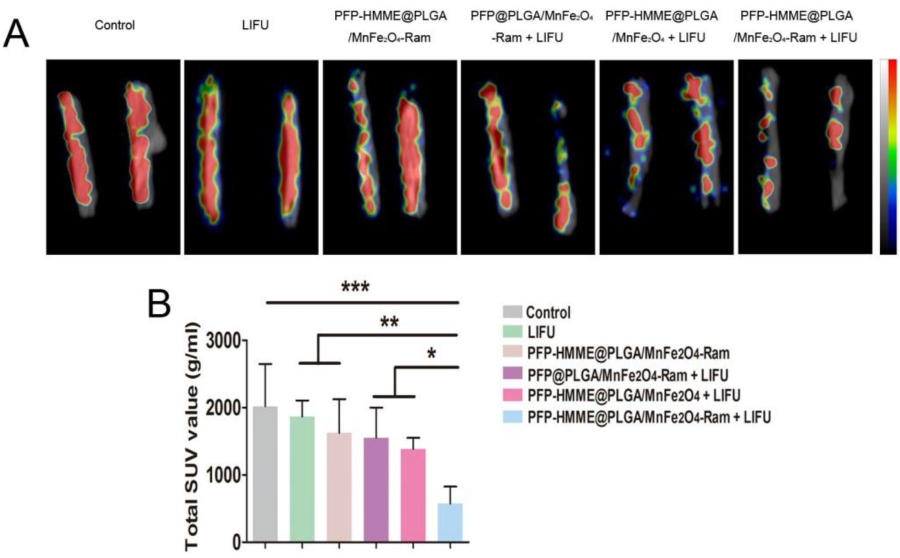

Figure S33. PFP-HMME@PLGA/MnFe$_2$O$_4$-Ram NP-mediated SDT decreases arterial inflammation in the rabbit femoral advanced plaque on day 28 after treatment. (A) Representative 3D fused ^{18}F-FDG positron emission tomography /computed tomography images of the rabbit femoral artery and **(B)** quantification of the total standardized uptake value (SUV) in the indicated groups (n = 4). Data are shown as the means ± SD. (B) ANOVA with Dunnett's post-hoc test. * $p < 0.05$, ** $p < 0.01$, *** $p < 0.001$.

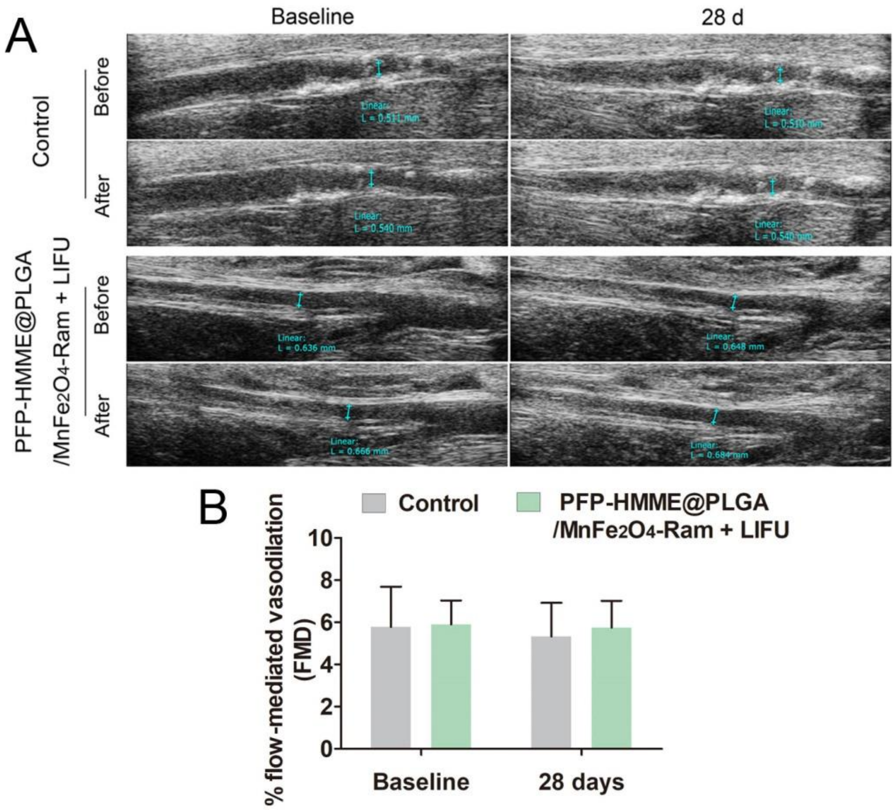

Figure S34. The effect of PFP-HMME@PLGA/MnFe$_2$O$_4$-Ram NP-mediated SDT on the endothelial cell function in the atherosclerotic plaque rabbits. (A) Representative ultrasound scans from femoral artery at baseline (left panels) and 28 days (right panels) after PFP-HMME@PLGA/MnFe$_2$O$_4$-Ram NP-mediated SDT, showing the artery at rest (before) and reactive hyperaemia (after). **(B)** Flow-mediated vasodilation quantification in the indicated groups (n = 4). Data are shown as the means ± SD. (B) two-way ANOVA with repeated measures with Tukey's post-hoc test.

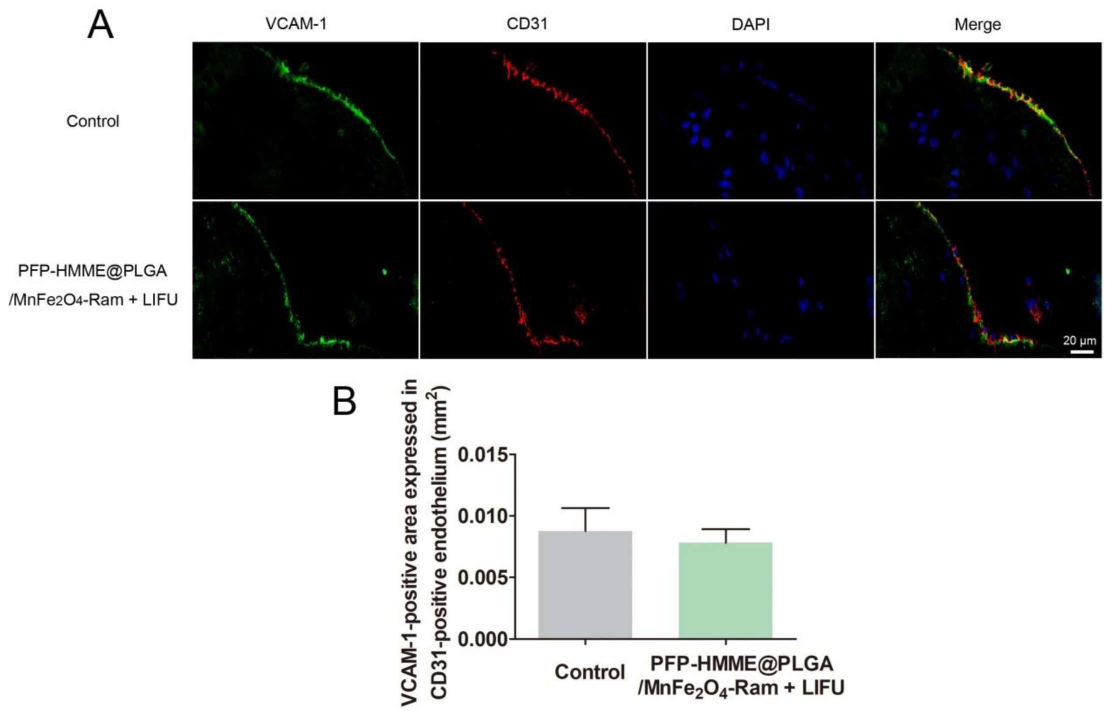

Figure S35. The effect of PFP-HMME@PLGA/MnFe$_2$O$_4$-Ram NP-mediated SDT on the VCAM-1-positive area expressed by the endothelium in the atherosclerotic plaque rabbits. At 28 days after treatment, the rabbit femoral artery cryosections were used for VCAM-1 and CD31 double immunofluorescence staining. **(A)** Representative immunofluorescence staining of plaque sections and **(B)** the quantification of VCAM-1-positive area expressed by the endothelium in the indicated groups (n = 4). Data are shown as the means ± SD. (B) Student's unpaired *t*-test.
